# Compositional complexity buffers free-volume sensitivity and serrated flow in metallic glasses

**DOI:** 10.1038/s41524-025-01933-7

**Published:** 2026-01-20

**Authors:** Anurag Bajpai, Jaemin Wang, Dierk Raabe

**Affiliations:** Max Planck Institute for Sustainable Materials, Düsseldorf, Germany

**Keywords:** Materials science, Physics

## Abstract

Processing history imprints metallic glasses (MGs), yet whether compositional complexity desensitizes structure and mechanics to quench rate remains unresolved. We use large-scale molecular dynamics along a controlled Cu-Zr complexity ladder, Cu_50_Zr_50_, Cu_47.5_Zr_47.5_Al_5_, and Cu_45_Zr_45_Al_5_Ti_5_, vitrified over 10^11^–10^15^ K·s^−1^ and probed by spherical nanoindentation. Additionally, composition-resolved Cu_*x*_Zr_100−*x*_ sweep (*x* = 40–65 at.%) and a microalloying series Cu_50-*z*/2_Zr_50-*z*/2_Al_*z*_, (*z* = 1–5 at.%) disentangle configurational entropy-driven effects from enthalpic and structural covariates. Atomic free volume is obtained from radical-Voronoi tessellation; non-affine rearrangements are quantified by Falk–Langer ${D}_{\min }^{2}$ field and clustered in three dimensions. Three quantitative descriptors capture the dispersion of free volume and its quench rate sensitivity as a function of compositional complexity. Increasing compositional complexity narrows free-volume distributions across quench rates and systematically reduces the fast-slow disparity. A two-axis reconciliation emerges: within binary Cu-Zr, configurational entropy peaks near equiatomic and minimizes rate sensitivity, whereas across alloy families (binary→ternary→quaternary), increased species diversity and size/enthalpy mismatch further suppress sensitivity. Structure-property co-variation is consistent: at fixed rate, hardness, modulus and elastic recovery increase, while serration density, STZ number density, and plastic-zone volume decrease. Radial-distribution metrics and indentation-induced icosahedral losses corroborate enhanced short/medium-range stability. Compositional complexity thus provides a quantitative lever for processing-tolerant, high-performance Cu-Zr-based MGs.

## Introduction

Metallic glasses (MGs) combine topologically disordered atomic arrangements with exceptional yield strength, elastic strain limit, and corrosion resistance, making them attractive for structural and functional applications^1,2^. The absence of crystalline slip systems, however, forces plastic deformation to localize into shear transformation zones (STZs), that can coalesce into shear bands and precipitate catastrophic failure^3^. At the heart of this process lies the free volume, *i.e.*, the excess atomic space that facilitates non-affine atomic rearrangements and governs STZ nucleation and activation^4,5^.

Free volume (FV) and its spatial dispersion in MGs depends on processing history, particularly the vitrification or quench rate. Rapid quenching produces loosely packed structures with relatively high free volume and structural heterogeneity, whereas slower cooling allows for structural relaxation and denser atomic packing^6^. This sensitivity is especially evident under nanoindentation, where orders-of-magnitude differences in quench rate manifest in varied hardness, serration statistics, elastic recovery, and energy dissipation^7,8^. While molecular dynamics (MD) studies have characterized such effects in binary systems such as Cu-Zr^9–11^, they often focus on a single alloy composition or a narrow quench rate window, limiting insights into the broader compositional-structural-mechanical space^12^.

Recent studies indicate that increasing compositional complexity, i.e., using additional principal elements and concomitant size/chemistry mismatch, can enhance glass-forming ability, raise crystallization barriers, stabilize short-range order (SRO) and promote structural stability via atomic size mismatch, chemical frustration, and increased configurational entropy^13–16^. Multicomponent and high-entropy MGs have shown higher SRO, enhanced icosahedral packing, and resistance to shear localization^17,18^. However, it remains unclear whether this compositional complexity can also suppress the sensitivity of free volume to quench rate, thereby stabilizing mechanical performance across different processing conditions. Existing experimental work on ternary/quaternary systems has hinted at such effects^19–21^, but without quantitative metrics or atomistically resolved mapping of the deformation process.

In this work, we bridge this gap through a systematic, large-scale MD study along a controlled complexity ladder: binary (Cu_50_Zr_50_)^22^, ternary (Cu_47.5_Zr_47.5_Al_5_)^23^, and quaternary (Cu_45_Zr_45_Al_5_Ti_5_)^24^ MGs, each quenched over a four order-of-magnitude rate window (10^11^–10^15 ^K·s^−1^) and probed by nanoindentation. Further, to isolate the role of compositional complexity from enthalpic and structural covariates, we augment this ladder with a composition-resolved Cu-Zr sweep (Cu_*x*_Zr_100−*x*_, *x* = 40–65 at.%) and a low-*x* Cu-Zr-Al microalloying series (Cu_50−*z*/2_Zr_50−*z*/2_Al_*z*_, *z* = 1–5 at.%). We introduce three new quantitative metrics, the Free Volume Heterogeneity Index (FVHI) that reflects fictive-state dependent dispersion rather than a local predictor of yielding, the Quench Rate Sensitivity Index (QRSI), and the Compositional Complexity Index (CCI), enabling us to explicitly link compositional diversity to structural stability and mechanical robustness. This framework explicitly separates two axes of “complexity”: (*i*) entropy-within-family, which tends to enhance rate sensitivity by strengthening the drive toward SRO/MRO, and (*ii*) component-count/mismatch complexity, which suppresses rate sensitivity via kinetic sluggishness and self-averaging of local motifs. By quantifying how CCI modulates the coupling between quench rate and both structure (FV dispersion, SRO signatures) and mechanics (serration density, stiffness, elastic recovery, plastic-zone volume), we establish a metric-based pathway to design MGs with processing-tolerant performance that extends beyond conventional glass-forming-ability heuristics.

## Results

### Quench rate effects on short-range order

**Fig. 1:**
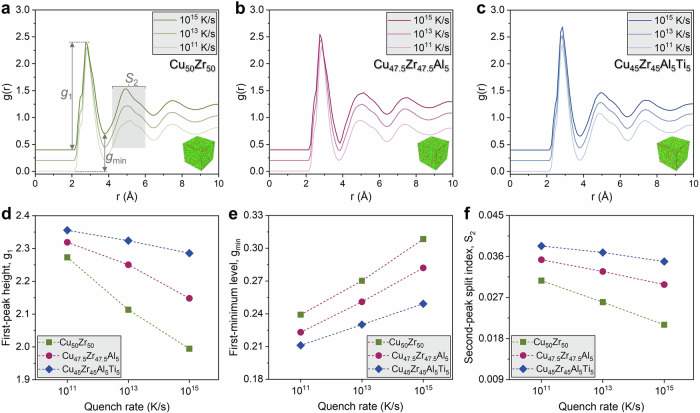
Quench rate and compositional complexity effect on the local structure of MGs. Total RDFs, *g*(r), for **a** Cu_50_Zr_50_, **b** Cu_47.5_Zr_47.5_Al_5_, and **c** Cu_45_Zr_45_Al_5_Ti_5_ quenched at 10^11^, 10^13^, and 10^15^ K·s^−1^. Annotated are the first-peak height *g*_1_, the first minimum *g*_min_, and the second-peak split window used to compute the split index *S*_2_. With slower quench (from 10^15^ → 10^11^ K·s^−1^), the first peak becomes taller/narrower, and the first minimum deepens, while the second-peak split becomes more pronounced, signatures of enhanced short- and medium-range order. Atomic configurations shown are representative snapshots at 10^11 ^K·s^−1^; **d**-**f** Quantitative trends extracted from *g*(*r*) versus quench rate for three compositions: (green squares) Cu_50_Zr_50_; (magenta circles) Cu_47.5_Zr_47.5_Al_5_; (blue diamonds) quaternary Cu_45_Zr_45_Al_5_Ti_5_. **d** First-peak height *g*_1_, **e** first-minimum level *g*_min_, and **f** second-peak split index ${S}_{2}=1-\frac{2{g}_{{val}}}{{g}_{2a}+{g}_{2b}}$ (with *g*_2a_, *g*_2b_ the two second-peak lobes and *g*_val_ the intervening valley).

Figure 1a–c shows the total radial distribution functions (RDFs), *g(r)*, for Cu_50_Zr_50_, Cu_47.5_Zr_47.5_Al_5_, and Cu_45_Zr_45_Al_5_Ti_5_ MGs quenched at 10^11^, 10^13^, and 10^15^ K·s^-1^. We quantify short- and medium-range order (SRO/MRO) with three physically interpretable scalars extracted from the RDFs: the first-peak height *g*_1_ (degree of nearest-neighbor packing), the first minimum *g*_min_ (depth of the cage surrounding the first shell), and a second-peak split index ${S}_{2}=1-\frac{2{g}_{{val}}}{{g}_{2a}+{g}_{2b}}$, which tracks the emergence of split subpeaks associated with fcc-/hcp-like motifs and icosahedral-related MRO (Fig. 1d–f; Tables S4 and S5). These RDF diagnostics are commonly used to monitor structural relaxation and packing efficiency in MGs during cooling and annealing^9,11,25^.

Across all alloys, faster quenching systematically weakens SRO (lower *g*_1_, higher *g*_min_, smaller *S*_2_). In Cu_50_Zr_50_, raising the quench rate from 10^11^ to 10^15 ^K·s^−1^ reduces *g*_1_ by 12.3%, lifts *g*_min_ by 18.8%, and suppresses *S*_2_ by 29.6%. The ternary Cu_47.5_Zr_47.5_Al_5_ is less sensitive; *g*_1_ is reduced by 7.4%, *g*_min_ rises by 12.0%, *S*_2_ lowers by 12.6%. The quaternary Cu_45_Zr_45_Al_5_Ti_5_ is the most buffered; Δ*g*_1_: -3.0%, Δ*g*_min_: +8.2%, Δ*S*_2_: -8.1%. Thus, quench rate-induced amplification of structural disorder is progressively damped as compositional complexity increases.

Further, for any given quench rate, complexity shifts the structure toward more efficient local packing and stronger cages as reflected in higher *g*_1_ and *S*_2_ and a lower *g*_min_ from binary to ternary to quaternary MGs. The same ordering holds at 10^13^ and 10^15 ^K·s^−1^. These monotonic trends indicate that adding Al (ternary) and Al+Ti (quaternary) stabilizes icosahedral-like and close-packed motifs during vitrification.

Mechanistically, the observed ordering follows from how chemical/size mismatch and multicomponent frustration reshape the energy landscape during vitrification. Additional principal elements broaden the distribution of pairwise interactions and strain-accommodation pathways, promote topological and chemical frustration and atomic size mismatch, which further favor dense local packing, elevating the energetic penalty for creating open, high-free-volume motifs and facilitating the nucleation and percolation of densely packed clusters (icosahedral-rich domains and close-packed fragments)^1,26^. Slower cooling deepens the first minimum and strengthens the first peak by allowing atoms to explore lower-energy basins, increasing icosahedral/close-packed SRO and medium-range connectivity^7,27^.

### Influence of compositional complexity on free volume evolution

We analyzed the distribution of the atomic-level free volume across all alloys to further evaluate how compositional complexity modulates the structural response to different quench rates. The free-volume landscape governs how amorphous metals accommodate strain: regions with above-average excess volume are softer, activate earlier under stress, and seed cascades of inelastic non-affine rearrangements (STZs). Quantitatively, increasing the quench rate by two orders of magnitude typically raises the specific free volume by ~0.5-1.5% (i.e., higher fictive temperature), broadens the Voronoi-volume distribution by ~20-40%, and reduces density by ~0.5-1.5%^1,3,5^.

**Fig. 2:**
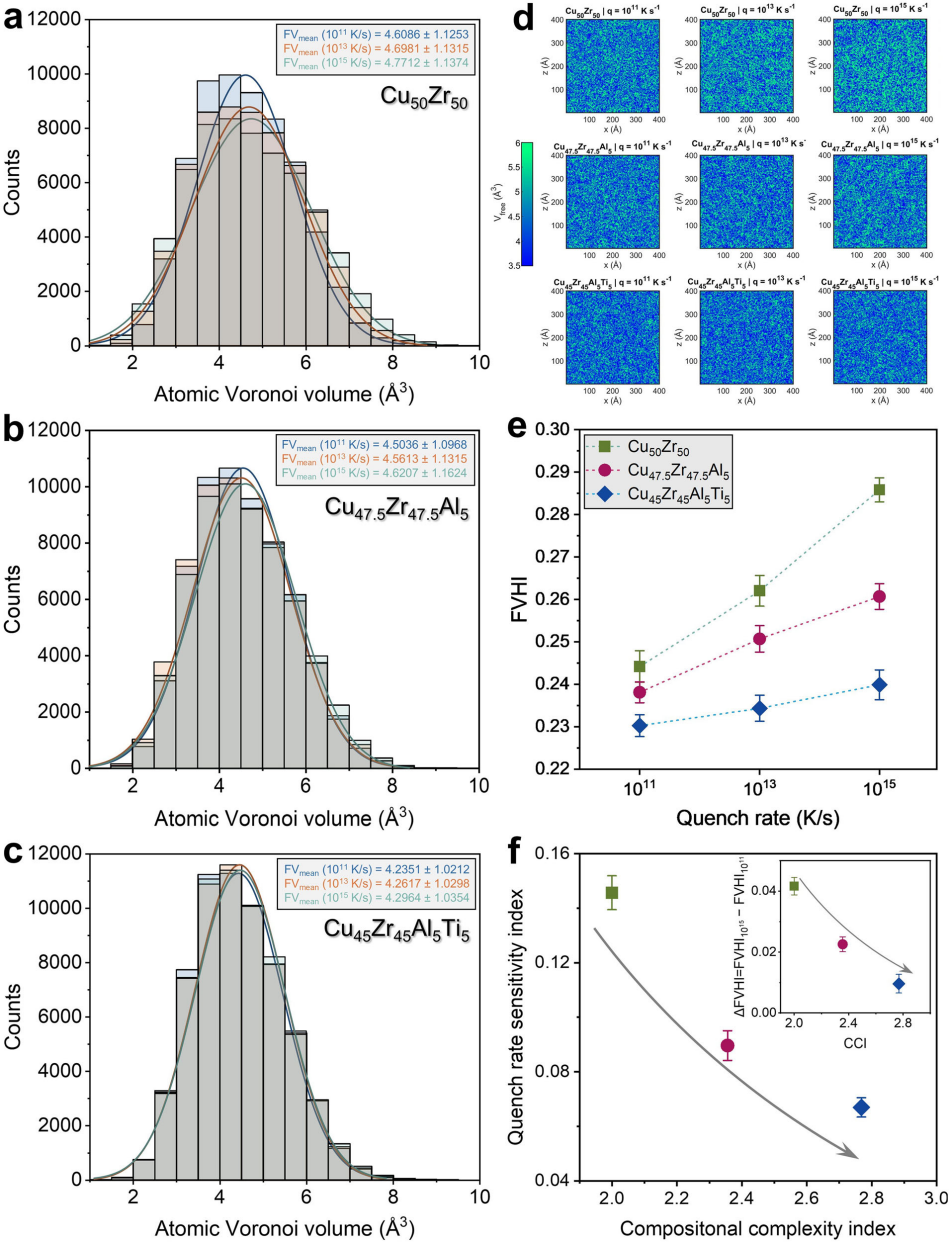
Free-volume statistics, spatial heterogeneity, and quench rate sensitivity. Distributions of atomic Voronoi volumes for **a** Cu_50_Zr_50_, **b** Cu_47.5_Zr_47.5_Al_5_, and **c** Cu_45_Zr_45_Al_5_Ti_5_ quenched at 10^11^, 10^13^, and 10^15 ^K·s^−1^. Volumes are obtained from radical Voronoi tessellation, showing progressive narrowing and reduced rate-sensitivity with increasing compositional complexity. Histograms (bins fixed across panels) are overlaid with kernel-density estimates. Faster quench broadens the distributions and shifts the mean to larger volumes; increasing compositional complexity narrows the distributions at all rates. **d** Spatial free-volume maps (same color scale across all panels) for each quench rate condition illustrating the progressive suppression of free-volume heterogeneity from binary → ternary → quaternary and from fast → slow quench. **e** Free-Volume Heterogeneity Index (FVHI = σ(*V*_free_)/〈*V*_free_〉) *vs*. quench rate, mean ± standard deviation (SD) over *N* = 5 independent seeds per condition; the binary alloy exhibits the steepest rise with rate. **f** Quench-Rate Sensitivity Index (${QRSI}=\,\frac{1}{{{FV}}_{{\dot{T}}_{\max }}}|\frac{\triangle FV}{\triangle {\log }_{10}\dot{T}}|$) of free volume plotted against Compositional Complexity Index (CCI = $\exp \left({\sum }_{i=1}^{n}{c}_{i}\mathrm{ln}\left({c}_{i}\right)\right)$, with *n* = number of present species). QRSI decreases monotonically with CCI, indicating that compositional complexity buffers the quench rate-induced amplification of free-volume heterogeneity. *Inset*: absolute change in free volume heterogeneity, $\varDelta {FVHI}={{FVHI}}_{{10}^{15}}-{{FVHI}}_{{10}^{11}}$, *vs*. CCI shows the same monotonic trend. Note that FVHI is a population-level descriptor of fictive-state dispersion.

To quantify not just the mean packing but its spatial dispersion, we evaluate the distribution of radical-Voronoi volumes and use the coefficient of variation, FVHI = *σ*(*V*_free_)/〈*V*_free_〉. The distributions of Voronoi volumes (Fig. 2a–c) broaden and shift to larger means with increasing quench rate, consistent with a higher fictive temperature and enhanced structural heterogeneity in rapidly vitrified MGs. Across alloys, FVHI increases with quench rate (Fig. 2e), but the growth is strongly alloy complexity dependent: for the binary Cu_50_Zr_50_ MG, it grows from 0.244 → 0.262 → 0.285 from 10^11^ to 10^15 ^K·s^−1^, reflecting the increase in fictive temperature and structural heterogeneity of rapidly vitrified MGs^28^. The same trend persists but is progressively muted as compositional complexity increases: Cu_47.5_Zr_47.5_Al_5_ shows 0.238 → 0.250 → 0.260, and Cu_45_Zr_45_Al_5_Ti_5_ is nearly rate-invariant (0.231 → 0.234 → 0.239). This attenuation is made quantitative by a QRSI, ${QRSI}=\,\frac{1}{{{FV}}_{{\dot{T}}_{max}}}|\frac{\Delta FV}{\Delta lo{g}_{10}\dot{T}}|$(*FV* being mean free volume and $\dot{T}$ is quench rate in K·s^-1^, see Supplementary Section S3 for details), which falls from 0.145 (binary) to 0.088 (ternary) to 0.064 (quaternary), Fig. 2f. This indicates that added chemical complexity progressively “pins” the glass against rate-induced disorder. Spatial maps (Fig. 2d) corroborate this: high-*V*_free_ patches become rarer and less contrasted as complexity increases.

For compositional complexity index (CCI), we adopt a thermodynamically grounded scalar: the exponential of the configurational entropy of mixing per mole normalized by *R*, CCI $\equiv \exp \left(\frac{{S}_{{config}}}{R}\right)=\exp (-{\sum }_{i}{c}_{i}\mathrm{ln}{c}_{i})$, with ${c}_{i}$ as the atomic fractions. This scaler carries a clear physical meaning as an extensive convex functional that penalizes compositional imbalance and is Schur-concave.

Plotting QRSI against CCI (Fig. 2f) yields a monotonic decay, establishing a quantitative link between compositional complexity and rate robustness of the free-volume field. The inset recasts the same information without any slope assumption by showing the absolute change in free volume heterogeneity, $\Delta {\rm{FVHI}}={{\rm{FVHI}}}_{{10}^{15}}-{{\rm{FVHI}}}_{{10}^{11}}$, which also decreases monotonically with CCI. Thus, both a differential (slope-based QRSI) and an integral (ΔFVHI) measure converge on the same conclusion: higher compositional complexity systematically suppresses the quench-rate-induced amplification of free-volume heterogeneity.

The suppression of free-volume heterogeneity by compositional complexity can be understood through two established mechanisms in multicomponent glasses. First, atomic-size mismatch and chemical frustration multicomponent Cu-Zr-(Al,Ti) glasses raise the energetic penalty for local dilatational fluctuations, narrowing the accessible distribution of *V*_free_^2,25^. Second, increasing species diversity raises the configurational entropy of mixing, fostering chemically mediated SRO/MRO, most notably icosahedral motifs, that are deep energy basins with intrinsically low excess volume and high shear resistance^7,27^. The prevalence and connectivity of these motifs grow with CCI, reducing the susceptibility of the free-volume landscape to thermal history.

These structural state descriptors (FVHI, QRSI) therefore provide the background against which the dynamic measures of non-affine activity are interpreted in subsequent discussion. It is important to note that FVHI is used as a state variable at the population level, a proxy for fictive temperature and structural dispersion, not as a local map for STZ nucleation. In the dynamic dilatancy view, the sites that will yield need not exhibit high static free volume at *t*=0; rather, they create sufficient excess volume under shear prior to activation^29^. Consequently, we couple FVHI with dynamic observables, serration density and ${D}_{\min }^{2}$-based STZ statistics, to interpret deformation.

### Mechanical response: hardness, serration statistics, and elastic recovery

**Fig. 3:**
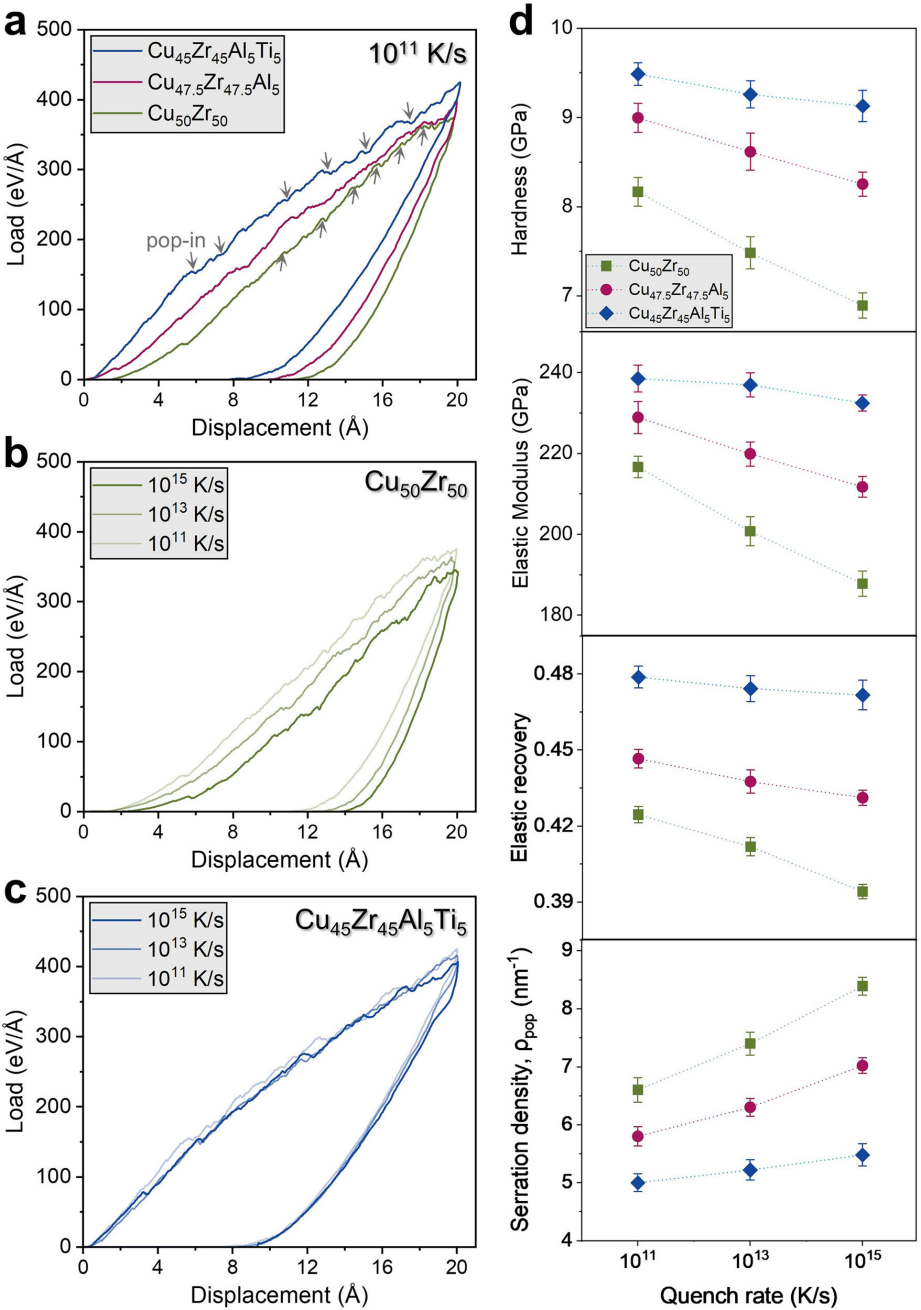
Nanoindentation response and serrated plasticity. **a** Representative load-depth curves at 10^11 ^K·s^−1^ for Cu_50_Zr_50_, Cu_47.5_Zr_47.5_Al_5_, and Cu_45_Zr_45_Al_5_Ti_5_ MGs indented with a spherical tip (*R* = 3 nm, *v* = 10 m·s^−1^) to a common depth of 2 nm. Arrows mark prominence-qualified pop-ins; Quench rate dependence of P-h for **b** Cu_50_Zr_50_ and **c** Cu_45_Zr_45_Al_5_Ti_5_, showing reduced serration and higher load with increased compositional complexity and slower cooling; **d** Indentation metrics *vs*. quench rate (mean ± SD over *N* = 5 seeds): hardness (H), elastic modulus (E), elastic recovery (ER) = (*h*_max_-*h*_res_)/*h*_max_, and serration density *ρ*_pop-in_ (events per nm of loading). Serration density is defined as the number of pop-ins per nanometer of indenter advance, counted over the plastic window 0.6 ≤ ℎ ≤ 2.0 nm (ℎ/*R* ≥ 0.2); pop-ins are detected as local minima in *dP*/*d*ℎ exceeding a data-adaptive prominence threshold with a minimum separation of 0.15 nm. E and H increase with alloy complexity and decrease with quench rate; ER follows the same ordering, whereas *ρ*_pop-in_ shows the opposite trend, confirming that compositional complexity suppresses rate-sensitive localized plasticity.

We probed the mechanical response via spherical nanoindentation with a 3 nm diameter rigid indenter to a fixed maximum depth of 2 nm at an approach velocity of 10 m/s. The displacement (P-h) curves in Fig. 3a–c for each alloy at different quench rates reveal several notable trends. Throughout, indentation properties were initially obtained from Oliver–Pharr unloading fits with excellent goodness-of-fit (Supplementary Fig. S2). The hardness and elastic modulus values were then corrected for pile-up around the indents using Meyer’s method. We reconstructed the post-indentation surface on a regular *x*-*y* grid and computed the Meyer hardness *H*_Meyer_ = *P*_max_/*A*_Meyer_ from the residual imprint area extracted by a statistical height threshold and light morphological denoising. Across alloys and rates (Supplementary Fig. S3), *H*_Meyer_ is systematically lower than *H*_Oliver-Pharr_, with the largest offsets in the fast-quenched binary glass and the smallest offsets in the quaternary Cu_45_Zr_45_Al_5_Ti_5_ MG. Quantitatively, the median (*H*_Meyer_ - *H*_Oliver-Pharr_)/H_Oliver-Pharr_ lies in the 3–10% range, peaking for Cu_50_Zr_50_ at 10^15^ Ks^−1^ and shrinking to within a few percent for Cu_45_Zr_45_Al_5_Ti_5_ at 10^11^ Ks^−1^.

At a fixed quench rate (Fig. 3a, 10^11^ K·s^−1^), the binary Cu_50_Zr_50_ shows the most pronounced serrated flow (frequent pop-ins), whereas the quaternary Cu_45_Zr_45_Al_5_Ti_5_ deforms relatively smoothly. In this work, we define the serration density, *ρ*_pop-in_, as the number of prominence-qualified pop-ins per nanometre of indenter advance, counted over a fixed plastic depth window 0.6 ≤ ℎ ≤ 2.0 nm (i.e., ℎ/*R* ≥ 0.2 for 3 nm spherical tip). The lower bound at ℎ = 0.6 nm coincides with the reproducible departure from elastic Hertzian scaling in our curves (fit misfit exceeds ~5% across all alloys/rates) and with the first statistically significant emergence of ${D}_{\min }^{2}$ clusters above the far-field *μ*+3*σ* threshold, i.e., the onset of irreversible non-affine activity rather than seating or elastic micro-noise. Pop-ins are identified as local minima in d*P*/dℎ, with a minimum separation of 0.15 nm between adjacent minima (set above the depth-axis jitter/noise floor and the typical width of single events in our data) to prevent double-counting of a single burst fragmented by numerical roughness while still resolving distinct bursts. Because serrations are transient stress relaxations, the associated counts/densities are dynamic readouts of dilatant non-affine activity. We therefore do not equate high static free volume with a higher local pop-in probability; instead, we use serration density as a direct measure of the system’s propensity to dynamically create free volume and trigger STZs under load^29^. Quantitatively, *ρ*_pop-in_ rises steeply with quench rate in every alloy, but its absolute level and rate sensitivity are both suppressed by compositional complexity: at 10^15 ^K·s^−1^, *ρ*_pop-in_ ≈ 8.39 ± 0.20 nm^−1^ (binary) → 7.02 ± 0.10 nm^−1^ (ternary) → 5.48 ± 0.18 nm^−1^ (quaternary). This hierarchy is consistent with the classic view that intermittent bursts originate from activation of STZs in regions of excess free volume; fewer and less correlated bursts are expected as packing becomes more constrained^1,3^. The same ordering is reflected in contact stiffness *S* from the unloading fits (Supplementary Fig. S2), which increases from the binary to the quaternary at a given rate, signaling a stiffer, more recoverable contact.

Figure 3b, c (quench rate series for binary and quaternary MG) shows that faster quenching lowers the terminal load at a given depth and increases the incidence of displacement bursts, both signatures of a looser, higher free-volume MG with a lower barrier for local rearrangements^1,30^. The rate sensitivity of hardness and modulus extracted in Fig. 3d (and summarized in Table 1) quantifies this: in Cu_50_Zr_50_, H drops by 16% and E by 13% when the quench rate is increased from 10^11^ to 10^15 ^K·s^−1^. The same change in the quaternary produces only −3.8% and −2.5%. Elastic recovery (ER) follows the same pattern, decreasing with increasing quench rate (more plasticity) but remaining the highest and least rate-sensitive in the quaternary (-1.5%), intermediate in the ternary (-3.4%) and lowest and most sensitive in the binary MG (-7.1%).

**Table 1:** Mean ± standard deviation values of hardness (GPa), elastic modulus (GPa), elastic recovery (ER), and shear-transformation zone (STZ) (counts per nm^3^) for three independent quenches (*N* = 5)

Alloy	Quench rate (K/s)	Hardness (*H*, GPa)	Elastic modulus (*E*, GPa)	ER (%)	STZ count (nm^−3^)
Cu_50_Zr_50_	1 × 10^11^	8.16 ± 0.098	216.64 ± 2.64	42.44 ± 0.32	36 ± 4
	1 × 10^13^	7.50 ± 0.111	200.71 ± 3.56	41.18 ± 0.36	75 ± 5
	1 × 10^15^	6.91 ± 0.102	187.78 ± 3.11	39.41 ± 0.28	110 ± 3
Cu_47.5_Zr_47.5_Al_5_	1 × 10^11^	8.99 ± 0.098	228.85 ± 1.97	44.65 ± 0.36	25 ± 4
	1 × 10^13^	8.61 ± 0.090	221.84 ± 1.89	43.75 ± 0.45	48 ± 3
	1 × 10^15^	8.25 ± 0.081	214.69 ± 1.62	43.11 ± 0.30	78 ± 5
Cu_45_Zr_45_Al_5_Ti_5_	1 × 10^11^	9.48 ± 0.129	236.47 ± 3.33	47.87 ± 0.43	21 ± 3
	1 × 10^13^	9.25 ± 0.153	234.87 ± 3.01	47.42 ± 0.51	35 ± 5
	1 × 10^15^	9.12 ± 0.176	229.41 ± 2.02	47.16 ± 0.58	49 ± 4

These mechanical hierarchies mirror the structural metrics established earlier: the FVHI increases with quench rate in all alloys, but the rise is steep in the binary (+16.8%) and shallow in the quaternary (+3.5%). Because indentation hardness and serration statistics are sensitive to the density and spatial dispersion of flow defects, the monotonic anti-correlation (H ↑/*ρ*_pop-in_ ↓/ER ↑) with FVHI ↓ across compositions is consistent with the STZ theory and prior nanoindentation studies on MGs^1,31^.

The composition dependence at a fixed quench rate is equally revealing. At 10^13 ^K·s^−1^, H ranks as quaternary (9.25 ± 0.153 GPa) > ternary (8.61 ± 0.090 GPa) > binary (7.50 ± 0.111 GPa), while *ρ*_pop-in_ ranks in the opposite order (5.22 ± 0.13 < 6.31 ± 0.09 < 7.42 ± 0.15 nm^−1^). The elastic modulus values show the same monotonic increase with increasing compositional complexity (234.87 ± 3.01 > 221.84 ± 1.89 > 200.71 ± 2.67 GPa) and ER follows suit (0.464 ± 0.005 > 0.437 ± 0.002 > 0.412 ± 0.003). These trends are consistent with the well-documented role of increased chemical/size disorder in promoting icosahedral SRO and more stable cages that resist shear localization, thereby elevating stiffness and reducing intermittent plasticity^7,9,25^. This stabilization is captured compactly by the configurational-complexity index CCI (increases from binary → ternary → quaternary) and by the QRSI computed from free-volume statistics; which decreases monotonically with increasing complexity (Fig. 2f), and the same ordering appears in the rate sensitivities of H, E, and ER (Fig. 3d). In other words, compositional complexity not only strengthens the glass but also desensitizes its mechanical response to thermal history, behavior consistent with contemporary reports on multi-principal-element metallic glasses that exhibit smoother (less serrated) plastic flow and enhanced damage tolerance attributable to chemically stabilized SRO^32–34^.

### Non-affine inelastic displacement and STZ mapping

To probe the atomic-scale deformation dynamics, we calculated the non-affine displacement (${D}_{\min }^{2}$) of atoms with respect to their pre-indentation configuration. ${D}_{\min }^{2}$ serves as a sensitive marker for detecting STZs and regions of localized irreversible deformation. Methodologically, using ${D}_{\min }^{2}$ as a local, frame-invariant measure of non-affine displacement directly identifies atoms participating in irreversible shear transformations, and clustering them in three dimensions provides a size- and number-based STZ census rather than a single global threshold count. Non-affine activity was quantified using the Falk–Langer ${D}_{\min }^{2}$ measure with a neighbor cutoff of 4.0 Å. To enable fair comparison across alloys and rates, the detection threshold was set relative to the far-field ${D}_{\min }^{2}$ distribution of each configuration (operationally, *μ*+3*σ* of atoms at distances >2.5 *R* from the contact), which removes baseline shifts caused by rate/composition and isolates state-comparable excursions. Active atoms were voxelized and 26-connected to define STZ clusters, from which we report cluster count (number density) and plastic-zone volume at matched indentation depth^35,36^.

**Fig. 4:**
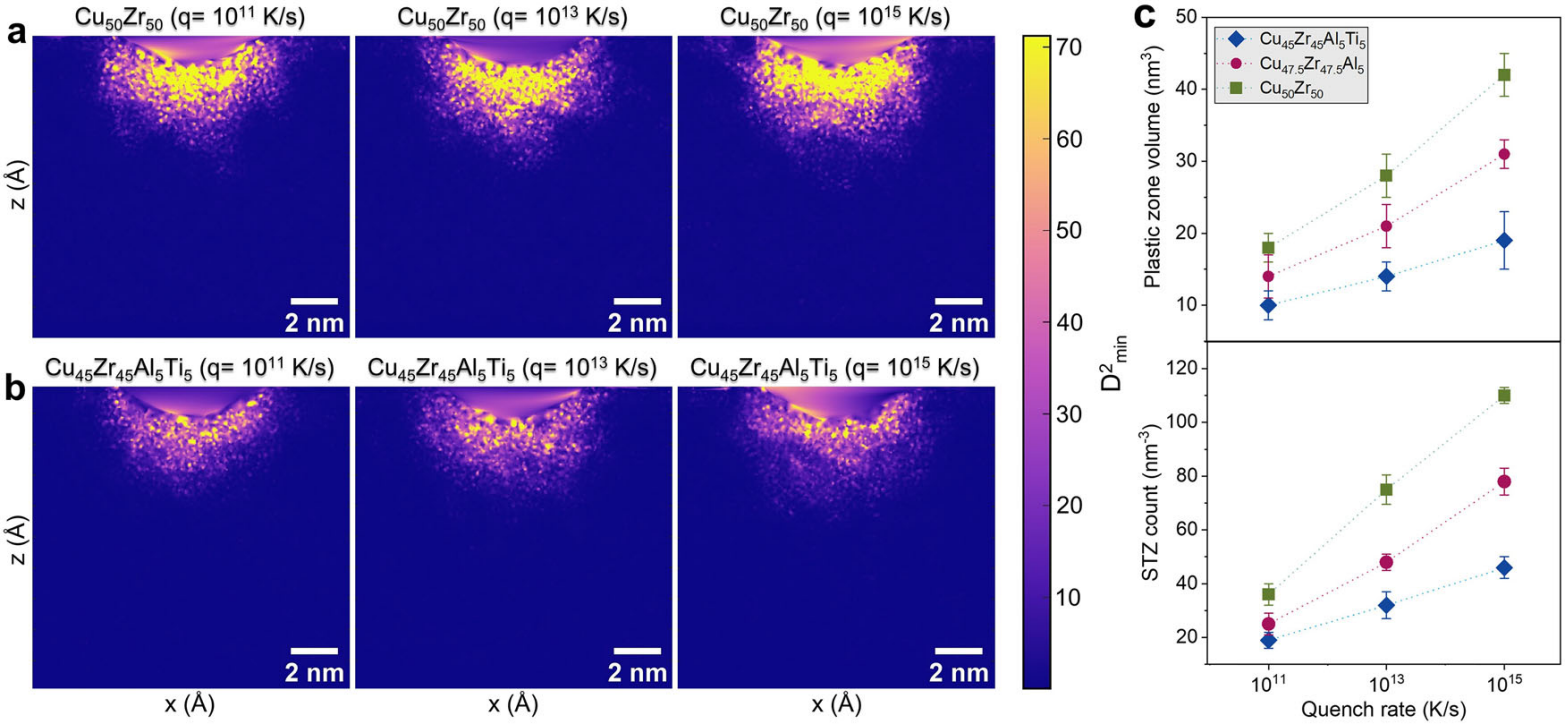
Deformation mechanisms under nanoindentation. Non-affine displacement (${D}_{\min }^{2}$) fields at maximum depth for **a** Cu_50_Zr_50_ and **b** Cu_45_Zr_45_Al_5_Ti_5_, for various quench rates. ${D}_{\min }^{2}$ active atoms are defined using a far-field referenced threshold (*μ*+3*σ*) in each configuration and clustered (26-connected) to obtain STZ number density and plastic-zone volume at matched depth (dynamic readouts of dilatant non-affine activity). The binary MG exhibits bright, localized lobes beneath the indenter, whereas the quaternary MG shows a smaller, more diffuse plastic zone. **c** Shear-transformation statistics *vs*. quench rate, mean ± SD over *N* = 5 seeds: STZ count (per nm^3^) and plastic-zone volume (nm^3^).

The non-affine strain fields under the indenter also resolve how processing history and composition set the length scale of irreversible rearrangements. These ${D}_{\min }^{2}$ fields and the derived STZ clusters capture the dynamic, dilatant rearrangements central to shear-transformation activation^29^, thus providing the dynamic complement to the population-level FVHI descriptor. Across all alloys, the ${D}_{\min }^{2}$ (Figs. 4a, b and S4) displays the canonical teardrop plastic zone beneath the contact with intense lobes oriented along the maximum-shear directions. With increasing quench rate, the bright ${D}_{\min }^{2}$ population thickens and extends deeper, indicating a higher density of localized atomic shuffles per unit volume. This trend is the atomistic counterpart of the Spaepen–Argon picture: faster quenching traps a higher fictive temperature and excess free volume, which lowers barriers for shear-transformation activation and promotes cascade percolation under indentation^37^.

Quantitatively, 3D voxel clustering of ${D}_{\min }^{2}$ shows a monotonic rate-dependence of STZ number density and plastic-zone volume, but with a pronounced suppression across the three quench rates as compositional complexity increases (Fig. 4c). For the binary glass, STZ density rises from 36 ± 4 to 110 ± 3 nm^−3^ as quench rate increases 10^11^ → 10^15 ^K·s^−1^; the corresponding plastic-zone volume grows from 18.2 ± 2.6 to 42.2 ± 3.1 nm^3^. The ternary shows a gentler rise (25 ± 4 → 78 ± 5 nm^−3^; 14.7 ± 3.6 → 31.2 ± 2.1 nm^3^), and the quaternary is most resistant (19 ± 3 → 46 ± 4 nm^−3^; 10.3 ± 2.2 → 19.4 ± 4.1 nm^3^). These real-space counts mirror the load-displacement statistics: serration density (ρ_pop-in_) tracks the same ordering (binary > ternary > quaternary at a given quench rate) and increases with quench rate, consistent with more frequent avalanches of STZ activity when the structure contains larger free-volume fluctuations^38^.

The ${D}_{\min }^{2}$ maps also reveal a composition-dependent localization width. In the binary glass, the high-${D}_{\min }^{2}$ lobes are contiguous and coarsen rapidly with quench rate, whereas in the quaternary MG, they remain granular and spatially intermittent even at 10^15 ^K·s^−1^. This suppression of spatial percolation is consistent with the established role of densely packed icosahedral motifs in elevating local shear resistance and disrupting the autocatalytic triggering of neighboring STZs. In situ/atomistic studies have shown that STZs preferentially nucleate in “soft-spot” regions with reduced icosahedral connectivity, and that increasing icosahedral order impedes the growth and coalescence of rearrangements into a shear band^39^. In our investigation, the rate-induced loss of icosahedra (Δ*f*_icosahedral_) scales with both STZ density and plastic-zone volume across the series (largest in the binary, smallest in the quaternary), aligning with those observations (Fig. 7).

Taken together, the maps and statistics indicate a clear structure-mechanics linkage: quench rate controls the activity of STZs through its effect on stored free volume. In contrast, compositional complexity controls the connectivity of STZs by stabilizing icosahedral SRO. The first raises the frequency of local non-affine events; the second frustrates their geometric coalescence. The outcome is a smaller, more diffuse plastic zone and fewer STZs per volume in the quaternary alloy at any given quench rate, accompanied by higher hardness, modulus and elastic recovery and reduced serration density, an integrated signature of a more stable deformation field^40^.

### Composition-resolved binary sweep (Cu_*x*_Zr_100−*x*_): structure and mechanics

We interrogated the binary backbone alloy system Cu_*x*_Zr_100−*x*_ (*x* = 40-65 at.%) to decouple composition-driven changes in chemical/topological order from the effects of number of components^41^. In a binary alloy system, the configurational entropy of mixing peaks at *x* = 50 at.% (Supplementary Fig. S5a). Combining this with the strongly negative Δ*H*_mix_ (Cu-Zr) and the pronounced atomic-size mismatch (*r*_Zr_ > *r*_Cu_) sets a well-defined metallurgical expectation: chemical short-range order (CSRO) and dense Zr-centered polyhedra (icosahedra and related close-packed motifs) are maximally stabilized close to the equiatomic composition and progressively weakened as the alloy becomes Cu- or Zr-rich^11,27^. The sweep therefore provides a stringent internal control for the hypothesis that “compositional complexity” buffers the structure against quench-rate perturbations.

The total RDFs, *g*(*r*), across the sweep (Supplementary Fig. S6) show the canonical signatures of structural relaxation with decreasing quench rate at every composition, namely, taller first peak (*g*_1_), deeper first minimum (*g*_min_), and a clearer second-peak splitting (*S*_2_), indicating enhanced nearest-neighbor packing, a stiffer cage surrounding the first shell, and stronger medium-range connectivity^42,43^. At fixed quench rate, increasing the Cu fraction monotonically raises *g*_1_, lowers *g*_min_, and increases *S*_2_ (Supplementary Fig. S8a–c), consistent with improved packing as the average metallic radius decreases and with the growing population of Zr-centered icosahedra stabilized by favorable Cu-Zr bonding^25,44^. Free-volume statistics extracted from radical-Voronoi tessellations corroborate these trends: the mean Voronoi volume contracts and the distribution narrows with increasing Cu (Supplementary Fig. S7), and the dispersion metric FVHI decreases correspondingly, evidencing a reduction of spatial dilatation heterogeneity (Supplementary Fig. S8d).

**Fig. 5:**
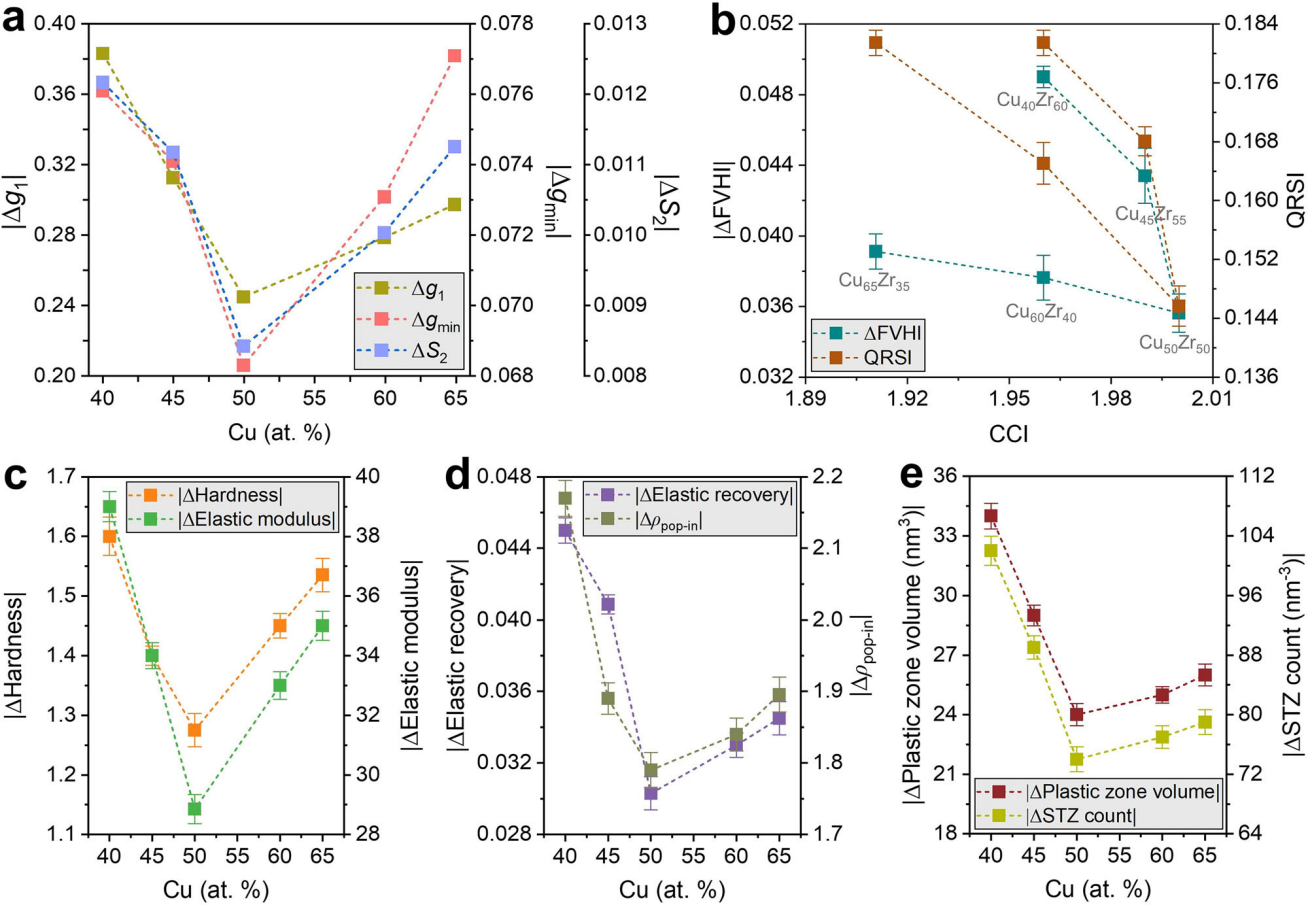
Cu-Zr composition sweep: rate-sensitivity landscape of structure and mechanics. **a** Rate-induced changes in short-/medium-range order from the total RDF: absolute fast-slow differences (10^15^–10^11^ Ks^−1^) of first-peak height ∣Δ*g*_1_∣, first-minimum level ∣Δ*g*_min_∣, and second-peak split index |ΔS_2_| for Cu_*x*_Zr_100−*x*_ (*x* = 40, 45, 50, 60, 65 at.%); **b** Structural heterogeneity metrics across the sweep: change in free-volume heterogeneity index ∣ΔFVHI∣ (left axis) and the quench-rate sensitivity index of mean free volume (QRSI; right axis) plotted against the CCI. Both ∣ΔFVHI∣ and QRSI exhibit a broad minimum near equiatomic composition, indicating maximal buffering of the fictive-temperature response at high compositional symmetry; **c** Mechanical rate sensitivities: absolute changes in hardness |Δ*H*| (left axis) and elastic modulus |Δ*E*| (right axis) from 10^11^ to 10^15^ K·s^−1^, mirroring the structural minimum at ~50 at.% Cu. **d** Dissipation/instability readouts: absolute changes in elastic recovery ∣ΔER∣ (left axis) and serration (pop-in) density |Δ*ρ*_pop-in_| (right axis) likewise minimize near equiatomic composition. **e** absolute changes in plastic-zone volume (left axis) and STZ count (right axis) extracted from ${D}_{\min }^{2}$ clustering; both track the same central minimum. Symbols give means over 5 independent seeds; error bars are 95% bootstrap CIs. Collectively, the sweep demonstrates a composition-resolved suppression of rate sensitivity that is strongest at equiatomic Cu-Zr, consistent with the peak in CCI and the concurrent stabilization of SRO/MRO and reduced free-volume dispersion.

The key finding is the rate sensitivity of these structural measures. When we compare the slow (10^11^ Ks^−^^1^) and fast (10^15 ^Ks^−1^) quench rates, |Δ*g*_1_|, |Δ*g*_min_|, and |Δ*S*_2_| all display a pronounced minimum at Cu_50_Zr_50_ and rise symmetrically toward both composition extremes (Fig. 5a). The same central minimum is observed for the free-volume response: the absolute change |ΔFVHI| and the quench-rate sensitivity index QRSI collapse at ≈ 50 at.% Cu and increase on either side (Fig. 5b). Metallurgically, this behavior is expected. Near equiatomic regime, the entropy maximum suppresses chemical clustering, enabling the liquid to more fully explore and populate the deep energy basins associated with dense Zr-centered polyhedra during vitrification^44^. The resulting MG forms with a lower fictive temperature and a more strongly connected icosahedral network; its local cages are already “tight,” hence, raising the quench rate perturbs *g*(*r*) features and the free-volume field only weakly^3,5,45^. As the composition drifts away from the 50/50 composition, CSRO weakens, the icosahedral basin depth and connectivity diminish, and the same change in quench rate produces a larger increase in excess volume and a larger degradation of SRO/MRO.

Mechanical responses measured by nanoindentation mirror these structural regularities. At a given composition, faster quenching reduces terminal load at fixed depth and increases serration incidence, enlarges the plastic zone, and raises the STZ count, consistent with a higher fictive temperature and a larger reservoir of shear-susceptible regions (Supplementary Figs. S9 and S10)^1,46^. At a fixed rate, hardness, elastic modulus, and elastic recovery increase with Cu content, while serration density, plastic-zone volume, and STZ number decrease, each trend tracking the reduction in FVHI and the strengthening of icosahedral connectivity with increasing Cu addition (Supplementary Fig. S11). Most importantly, the quench rate-induced changes in all mechanical metrics exhibit the same V-shaped composition dependence as the structural metrics: |ΔH|, |ΔE|, |ΔER|, |Δ*ρ*_pop-in_|, |ΔPlastic zone volume|, and |ΔSTZ size| are minimized at or very near Cu_50_Zr_50_ and grow toward both ends of the composition sweep (Fig. 5c–e). ${D}_{\min }^{2}$ maps visualize this: the equiatomic glass shows the most compact, least rate-sensitive plastic zone; Cu- and Zr-rich glasses show broader, more rate-responsive zones (Figs. 4a and S10).

Taken together, the binary composition sweep of the Cu_*x*_Zr_100-*x*_ MG system establishes a rigorous, metallurgically consistent picture. Within a fixed species set, the equiatomic composition, where *S*_config_ is maximal and Δ*H*_mix_ and size mismatch jointly favor dense CSRO, produces a glass with low free-volume heterogeneity, high icosahedral content, and a structurally “stiff” energy landscape. That landscape is intrinsically less sensitive to changes in fictive temperature, so quench-rate perturbations translate into smaller variations in both structure and mechanics. Departures from the equiatomic composition ratio reduce chemical mixing and icosahedral stability, amplify the rate-induced growth of excess volume and cage softness, and thereby increase the sensitivity of hardness, modulus, recovery, serration statistics, and STZ activity. This composition-resolved analysis reconciles with the complexity ladder: maximizing chemically mixed, densely packed motifs, either by approaching equiatomic compositions or by increasing number of components while maintaining favorable Δ*H*_mix_ and size mismatch, systematically suppresses quench-rate sensitivity and yields processing-tolerant metallic glasses.

### Entropy-focused microalloying (Cu-Zr-Al): structure–mechanics coupling

To interrogate the specific role of configurational complexity while minimally perturbing the binary Cu-Zr backbone, we introduced dilute Al additions (1-5 at.%) at Cu:Zr ≈ 1:1. This protocol yields a monotonic rise in the normalized configurational entropy, *S*_config_ (Supplementary Fig. S5b), in contrast to the non-monotone variation along the binary Cu-Zr sweep (Supplementary Fig. S5a), thereby providing a clean handle on entropy-driven effects^47^.

**Fig. 6:**
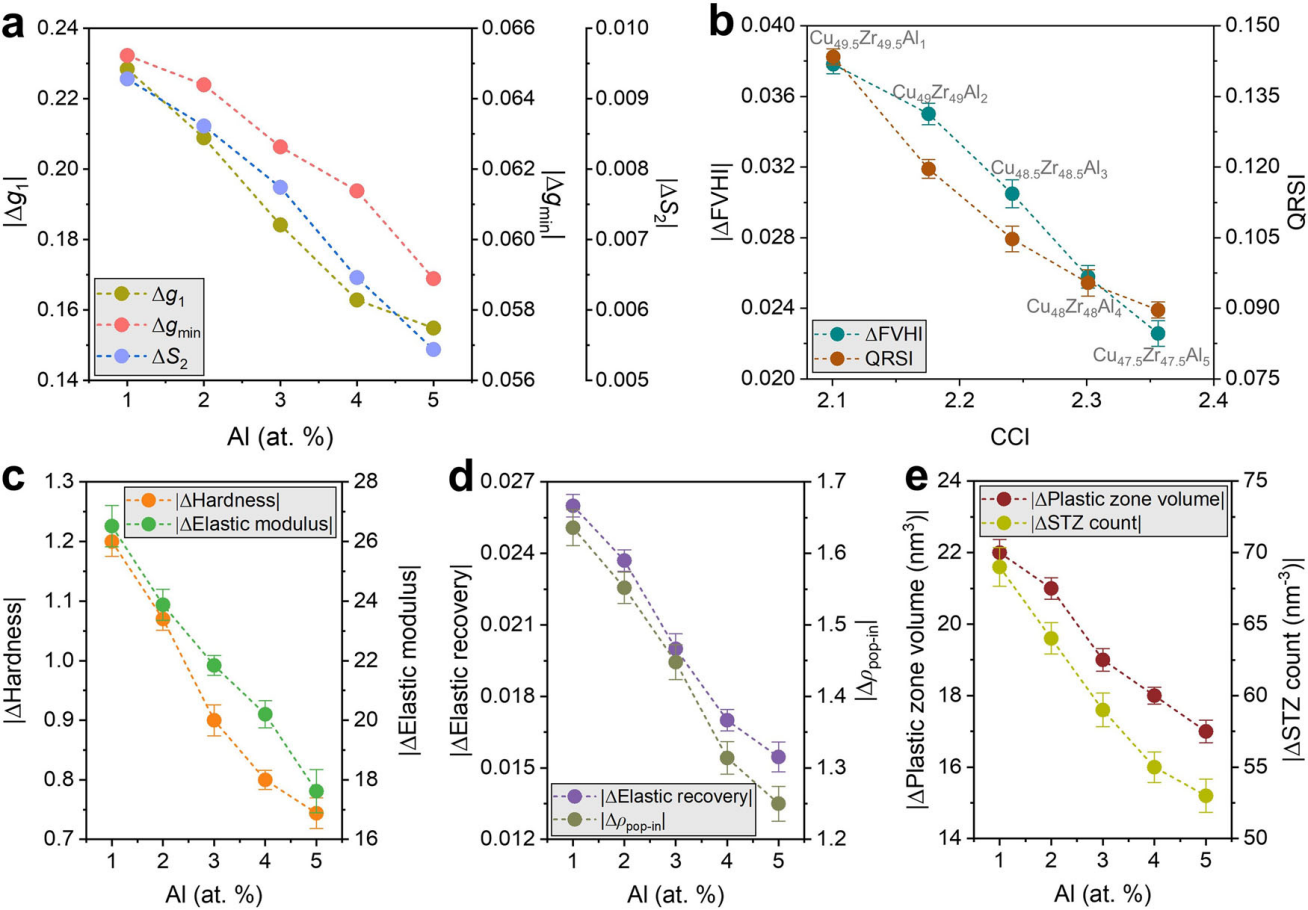
Al microalloying of Cu–Zr: entropy-focused suppression of rate sensitivity. **a** Rate-induced structural changes from total RDFs for Cu_50−*z*/2_Zr_50−*z*/2_Al_*z*_ (*z* = 1-5 at.%): absolute fast–slow differences of first-peak height ∣Δ*g*_1_∣, first-minimum level ∣Δ*g*_min_∣, and second-peak split index ∣Δ*S*_2_∣. All three decrease monotonically with Al, indicating increasingly stable short-/medium-range order; **b** Structural heterogeneity metrics vs compositional complexity index (CCI; normalized mixing entropy): change in free-volume heterogeneity ∣ΔFVHI∣ (left axis) and quench-rate sensitivity of mean free volume (QRSI; right axis). Both decline systematically with CCI, evidencing entropy-driven buffering of fictive-temperature effects; **c** Mechanical rate sensitivities: absolute changes in hardness *|*Δ*H|* (left axis) and elastic modulus ∣Δ*E*∣ (right axis) between 10^11^ and 10^15 ^K·s^−1^; sensitivity diminishes with Al addition; **d** Dissipation/instability readouts: absolute changes in elastic recovery ∣ΔER∣ (left axis) and serration (pop-in) density ∣Δ*ρ*_pop-in_∣ (right axis), both decreasing with Al; **e** absolute changes in plastic-zone volume (left axis) and STZ count (right axis), showing monotonic suppression with microalloying. Symbols give means over independent seeds; error bars are 95% bootstrap CIs.

Structurally, the total pair correlations preserve the Cu-Zr first-shell topology but exhibit systematic sharpening of the first maximum *g*_1_, deepening of the first minimum *g*_min_, and growth of the second-peak split index *S*_2_ with increasing Al at any fixed quench rate (Supplementary Fig. S12a–d). These signatures indicate higher near-neighbor packing efficiency and strengthened MRO^11,27^. Consistently, the radical-Voronoi statistics show a progressive contraction of both the mean free volume and its dispersion with Al at all quench rates (Supplementary Fig. S13a–d), which is captured by a monotone decrease of the heterogeneity index FVHI and, crucially, a marked attenuation of its rate-induced change (Supplementary Fig. S14a–d). Quantification via the quench-rate sensitivity index, QRSI, demonstrates a near-linear decrease with the CCI across the series (Fig. 6b), establishing that increased elemental diversity “pins” the fictive-temperature response of the free-volume field^43,44^.

The mechanical response follows the same hierarchy. Representative P-h curves show higher contact load and visibly fewer displacement bursts with Al at fixed quench rate (Supplementary Fig. S15a–d), while matched-depth ${D}_{\min }^{2}$ fields reveal a contracted plastic zone and reduced non-affine activity (Supplementary Fig. S16). Aggregated metrics (Supplementary Fig. S17) demonstrate monotonic increases of hardness *H*, indentation modulus *E*, and elastic recovery (*ER*) with Al, accompanied by decreases in serration density *ρ*_pop-in_, plastic-zone volume, and STZ count. The fast-slow deltas (10^15^ → 10^11 ^K s^−1^) of all metrics shrink with increasing Al, i.e., rate sensitivity is systematically buffered. Figure 6 summarizes these results: |Δ*g*_1_∣, ∣Δ*g*_min_∣, and ∣Δ*S*_2_∣ fall monotonically with Al (Fig. 6a); ∣ΔFVHI∣ and QRSI decrease coherently with CCI (Fig. 6b); and the corresponding mechanical deltas, ∣Δ*H*∣, ∣Δ*E*∣, ∣Δ*ER*∣, ∣Δ*ρ*_pop-in_∣, ∣Δplastic-zone volume∣, and ∣ΔSTZ count∣, exhibit the same monotone attenuation (Fig. 6c–e).

These trends are metallurgically consistent with the coupled action of CSRO and topological/geometric frustration. First, introducing Al increases the atomic-size-mismatch variance relative to the binary mean, suppressing high-dilatation outliers and narrowing free volume distributions; the concomitant deepening of *g*_min_ and reduction of FVHI follow directly^11,27,43^. Second, Al exhibits favorable (negative) mixing enthalpies with both Cu and Zr and a strong propensity to stabilize icosahedral-like CSRO within Cu-Zr matrices^25,27^. The observed growth in *S*_2_ and the contraction of plastic zone volume with Al are consistent with an increased density of deep MRO basins, an elevated local shear-activation barrier, and weaker spatial correlations among STZ nucleation sites^44,48^. Because quench-rate effects predominantly act via fictive-temperature elevation, i.e., inflation and spatial biasing of free volume, the Al-induced homogenization of the free-volume field provides a direct mechanistic route to the observed reductions in FVHI-, QRSI-, and mechanics-based rate sensitivity (Fig. 6). In summary, entropy-focused microalloying in Cu-Zr-Al produces a stiffer, stronger, and more elastically recoverable glass whose structural state becomes substantially less susceptible to processing history with increasing microalloying towards its equiatomic composition space.

### Integrating structure and mechanics: a complexity-controlled pathway

Integrating all observables across MG chemistry and processing reveals a single, complexity-controlled pathway that links structural state, local kinematics, and macroscopic response in Cu-Zr-(Al,Ti) metallic glasses. Faster quenching elevates the fictive temperature and increases excess volume, while added chemical diversity (higher CCI) counteracts this effect by enhancing packing efficiency through atomic-size mismatch and chemical frustration. The resulting statistics of the free-volume field, captured by the distribution of radical-Voronoi volumes and its heterogeneity index FVHI, therefore broaden strongly with quench rate in the binary, moderately in the ternary, and only weakly in the quaternary glass (Fig. 2a–e). Quantifying this attenuation with the quench-rate sensitivity index QRSI demonstrates a monotonic decrease with CCI (Fig. 2f), i.e., compositional complexity buffers the structure against quench rate-induced disorder.

These statistical differences map directly onto the short- and MRO. In the binary Cu-Zr composition sweep, the first-peak height of the RDF is lower and the first-minimum level higher, consistent with looser nearest-neighbor packing; both metrics improve with added Cu up to ≈50 at.% and then degrade as Cu is further increased, reflecting the well-known optimum in Cu-Zr icosahedral network formation (Supplementary Figs. S6–S8). Microalloying with Al steepens the RDF first peak, deepens the first minimum, and increases the split index of the second peak across all rates (Supplementary Figs. S12–S14), indicating strengthened SRO and more pronounced medium-range connectivity. Pre-indentation icosahedral clusters fractions follow the same hierarchy, largest in Cu_45_Zr_45_Al_5_Ti_5_, intermediate in Cu_47.5_Zr_47.5_Al_5_, smallest in Cu_50_Zr_50_, and decrease with quench rate (Supplementary Figs. S18 and S19). Importantly, the damage incurred by indentation, Δ*f*_icosahedral_, is minimal in the chemically complex glasses and maximal in the binary, signifying a more resilient icosahedral backbone at higher CCI (Supplementary Figs. S18–S21).

**Fig. 7:**
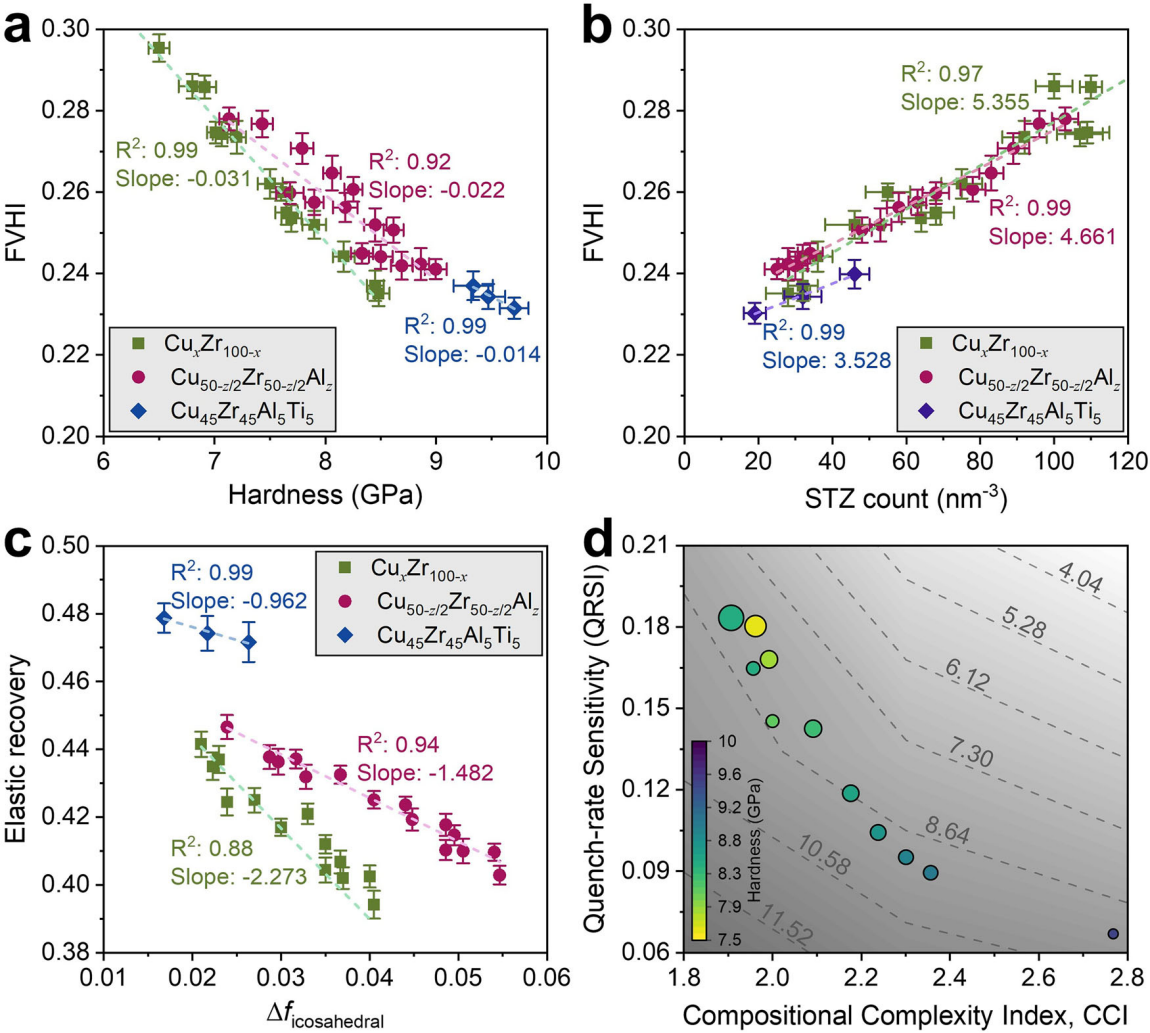
Short-range order and structure-property links. **a** Free-volume heterogeneity (FVHI) versus hardness, *H*, for the Cu-Zr sweep (green squares), the Al-microalloying series (magenta circles), and the quaternary MG (blue diamonds). Each symbol is mean ± 95% CI over seeds; lines are total-least-squares fits. FVHI anti-correlates with *H* within every family (slopes shown on panels), indicating that denser, more homogeneous glasses are harder; **b** FVHI versus STZ number density (from ${D}_{\min }^{2}$ clustering at matched depth). A strong positive correlation holds across all chemistries (family-specific slopes and *R*^2^ shown), linking larger free-volume dispersion to a higher density of plastic carriers; **c** Elastic recovery, ER, versus the loss of icosahedral order during indentation, Δ*f*_icosahedral_ = *f*_pre-indentation_ – *f*_post-indentation_. ER decreases as more icosahedra are destroyed, with the weakest sensitivity in the quaternary—consistent with its greater SRO stability; **d** Processing-tolerance/design map: quench-rate sensitivity of free volume (QRSI) plotted against compositional complexity index (CCI). Points mark alloy-rate conditions; color encodes hardness (GPa) and scatter size encodes FVHI at 10^11^ Ks^−1^. Gray dashed isolines are iso-hardness contours obtained from a smooth surface fit. The map reveals a clear design quadrant, high CCI/low QRSI, where metallic glasses are both hard and minimally quench rate-sensitive.

These kinematic signatures are mirrored one-for-one in the indentation response. At fixed rate, hardness *H* and modulus *E* scale with complexity (quaternary > ternary > binary) and decrease with increasing quench rate; elastic recovery *ER* shows the same hierarchy but the opposite rate trend (Figs. 3d, S11 and S17). This is consistent with the notion that tightly packed, icosahedrally enriched regions store, and recover, more elastic energy before irreversible rearrangement^1,5^. The intermittency encoded in the loading branch, serration (pop-in) density, defined as the number of statistically significant load-drops per nanometer of advance to *h*_max_ = 2 nm, rises with quench rate yet is consistently suppressed by complexity (Fig. 3a–d). The binary MG therefore carries the largest plastic-zone volumes, the highest STZ densities, and the most frequent pop-ins, in exact correspondence with its larger FVHI; the quaternary MG shows the converse. Cross-plots consolidate these couplings: FVHI anti-correlates tightly with hardness, and correlates with STZ density (Fig. 7a, b); ER decreases approximately linearly with Δ*f*_icosahedral_ (Fig. 7c), indicating that retention of icosahedral SRO under load promotes recoverable elasticity and suppresses shear localization, consistent with atomistic and experimental observations that icosahedral backbones screen STZ nucleation and limit shear band percolation^4^.

Kinematically, the non-affine displacement field ${D}_{\min }^{2}$ records the ease with which the solid accesses local plastic rearrangements. Consistent with Falk–Langer shear-transformation theory, the binary MG hosts larger plastic caps with dense clusters of high ${D}_{\min }^{2}$, whereas the quaternary exhibits compact, shallow caps with sparse hotspots at the same depth and rate (Figs. 4a, b, S10, S11, S16 and S17 for composition and microalloying series). Three-dimensional voxel clustering of active atoms converts these maps into physically interpretable measures: plastic-zone volume and STZ number density are highest in the binary, intermediate in the ternary, and lowest in the quaternary, and all increase with quench rate (Figs. 4c, S10, S11, S16 and S17). In Falk–Langer terms, added elements raise the energetic/entropic cost to reach the critical non-affine strain within a given volume, reducing the number and the lateral growth of rearranging clusters^7,12^. Interpreted in the dilatancy framework, higher complexity narrows the distribution of accessible dilatant pathways (lower FVHI, lower QRSI), so that larger stress excursions are required to dynamically create the critical free volume for STZ activation, reducing both STZ count and pop-in density at a fixed deformation state.

Finally, we place these relationships on a materials-design map. Using CCI as a chemistry-only abscissa and QRSI as a processing-sensitivity ordinate yields a smooth, monotonic trajectory: increasing compositional complexity moves the glass down and to the right, toward low structural heterogeneity and weak rate sensitivity (Fig. 7d). Superimposed iso-hardness contours in this space reveal that complexity not only stabilizes the structure against quench-rate perturbations (low QRSI) but also elevates absolute performance (higher *H*). In operational terms, alloys with higher CCI occupy regions of low FVHI, low STZ density, few pop-ins, small plastic zones, high ER, high H, and high E. The mechanistic picture is therefore internally consistent across statistics (FVHI, QRSI, CCI), structure (RDF metrics, Voronoi/icosahedral content), kinematics (${D}_{\min }^{2}$ maps, STZ counts, plastic-zone size), and mechanics (*H*, *E*, *ER*, serration density): compositional complexity reduces fictive-volume heterogeneity and raises the energetic and entropic barriers for non-affine rearrangement, thereby fragmenting STZ percolation and hardening the amorphous alloy while rendering its properties less sensitive to processing history.

It is important to note that all analyzes were conducted on as-quenched structures without post-quench annealing or structural relaxation. While this choice isolates the intrinsic influence of quench rate and composition on structural disorder, annealing may further modify the free volume landscape and STZ activity. Future investigations could examine how compositional complexity impacts the relaxation kinetics and enthalpy evolution during thermal aging. Within the present scope, however, the agreement presented substantiates a robust, complexity-controlled pathway for stabilizing and strengthening Cu-Zr-based metallic glasses, with CCI providing a succinct, defensible design coordinate that unifies composition, processing sensitivity, and performance.

### Relevance of simulation rates to experiments

It is critical to note that molecular dynamics necessarily employs quench rates of ≈10^11^–10^15 ^Ks^−1^ (here), and high loading velocities compared to laboratory experiments to access aging- and plasticity-relevant processes on nanosecond timescales, but the physics of plasticity in MGs implies that these choices translate absolute magnitudes rather than invert composition-dependent orderings or the structure-response links we report. Plastic flow in MGs is mediated by cooperative shear-transformation events drawn from an intrinsic barrier distribution and its structural state. Classic free-volume and STZ theories, therefore predict logarithmic/power-law rate effects that shift the stress scale while preserving trends tied to the underlying barrier statistics (i.e., chemistry and structural state)^3,5^. Consistently, the Johnson–Samwer relation shows a universal ($T/{T}_{g}$)^2/3^ reduction of the flow stress, with strain-rate entering as a weak activation term, implying that rate changes re-level the ordinate while preserving alloy rankings at comparable structural states^30^.

For indentation, the pertinent control parameter is the dimensionless indentation strain rate ${\dot{\varepsilon }}_{{ind}}\approx \dot{h}/h$, widely used in instrumented indentation. All curves in Fig. 3 are compared at matched deformation states (same *h*/*R*, same maximum depth), which ensures like-for-like kinematics even when h ˙is large in MD^49^. Rate-change and dynamic loading studies further show that serrated flow and pop-in activity persist across orders of magnitude in rate with modest sensitivity (small *m*), in line with an activation picture rather than a kinematic artifact^50^.

On the structural side, quench rate primarily sets the fictive temperature ${T}_{f}$ (and thus free-volume/packing); experiments and simulations alike show that mechanical response collapses when compared at matched structural state (e.g., similar $T/{T}_{g}$ or fictive state). This internal consistency is also observed across our three MD quench rates: absolute hardness/modulus shift with rate, but the alloy ordering and the structure–mechanics couplings (FVHI ↔ *H*, *E*, *ER* and STZ metrics) remain invariant^4,51,52^.

Together, these considerations and checks justify our comparative interpretation: while MD rates change absolute magnitudes, the trend fidelity, namely, that increasing compositional complexity buffers quench-rate sensitivity in both structure and mechanics, persists under experimentally relevant conditions.

## Discussion

Using large-scale MD across a Cu-Zr complexity ladder (Cu_50_Zr_50_ → Cu_47.5_Zr_47.5_Al_5_ → Cu_45_Zr_45_Al_5_Ti_5_) and three decades of quench rate (10^11^–10^15^ K·s^−1^), we establish a quantitative structure-mechanics link demonstrating that compositional complexity systematically suppresses quench-rate sensitivity while strengthening and stiffening the metallic glass. Structural disorder, characterized by the FVHI, decreases monotonically with added elements at every rate, and the Quench-Rate Sensitivity Index (QRSI) decays with the compositional complexity, indexed by CCI. The same ordering propagates to mechanics: at fixed rate the quaternary MG is consistently the hardest, stiffest, and most elastic-recoverable, while exhibiting the fewest displacement serrations, the smallest ${D}_{\min }^{2}$ plastic caps, and the lowest STZ densities. Indentation-induced loss of icosahedral SRO (Δ*f*_icosahedral_) follows the same hierarchy, being minimal in the quaternary and maximal in the binary, directly tying resilience of icosahedral backbones to recoverable elasticity and suppressed shear localization.

Two targeted composition sweeps explain how chemistry controls processing tolerance. First, a Cu–Zr binary sweep (Cu_*x*_Zr_100-*x*_, *x* = 40-65 at.%) shows that approaching equiatomic composition minimizes rate sensitivity of both structure (|ΔFVHI|) and mechanics (|Δ*H*|, |Δ*E*|, Δ*ER*), and reduces serration density, plastic-zone volume, and STZ counts. Thus, optimal binary packing, not absolute Cu or Zr content, governs resistance to fictive-temperature perturbations. Second, an entropy-focused microalloying series (Cu_50-*z*/2_Zr_50-*z*/2_Al_*z*_, *z* = 1-5 at.%) demonstrates that modest increases in configurational entropy produce a monotonic contraction of the free-volume landscape and a marked drop in QRSI, accompanied by higher *H*, *E*, and *ER* and lower serration density, plastic-zone size, and STZ density. Mechanistically, increasing complexity through composition perturbations towards equiatomic space or added elements deepen icosahedral basins, suppress long-tail free-volume fluctuations, and raise the barrier for STZ nucleation and coalescence.Taken together, the FVHI/QRSI/CCI framework thus provides a quantitative, transferable design rule: increasing compositional complexity yields processing-tolerant metallic glasses with stabilized deformation fields.

## Methods

### Molecular dynamics model systems

Classical molecular dynamics (MD) simulations were performed with LAMMPS to investigate glass formation and spherical nanoindentation in three model alloys: Cu_50_Zr_50_, Cu_47.5_Zr_47.5_Al_5_, and Cu_45_Zr_45_Al_5_Ti_5_^53^. Additionally, composition sweeps on the Cu_*x*_Zr_100-*x*_ (*x* = 40, 45, 50, 60, 65 at.%) system as well as microalloying series for Cu_50-*z*/2_Zr_50-*z*/2_Al_*z*_ (*z* = 1–5 at.%) system were also investigated. Initial configurations were generated on an fcc lattice (lattice parameter *a* = 3.7 Å) in a cubic box of dimensions 25 × 25 × 25 (62,500 atoms) with periodic boundary conditions.

### Interatomic potentials and parameterization

Interatomic interactions were described using the second-nearest-neighbor modified embedded atom method (2NN MEAM)^54^. Published parameter sets were adopted for the unary elements (Al, Cu, Ti, Zr) and for the binaries Al-Cu, Al-Ti, Al-Zr, Ti-Cu, and Zr-Cu^55–59^. Because no Zr-Ti potential was available, we developed a 2NN-MEAM Zr-Ti parameterization and validated it against DFT and experiment; the formalism and LAMMPS files are provided in Supplementary Sections S1 and S2, with parameter sets (Tables S1 and S2), intermetallic energetics and lattice constants (Table S3), and solid-solution lattice trends (Fig. S1). Ternary parameterizations not available in the literature were constructed following the procedure of Choi et al. for each ternary (*i*-*j*-*k*)^60^. These parameters were assigned mechanically derived values using an averaging strategy consistent with 2NN MEAM and validated for continuity at the binary/ternary limits. The resulting set completed the Al-Cu-Ti-Zr quaternary potential. All LAMMPS parameter files are provided in the Supplementary Information. A radial cutoff of 5 Å was used in all simulations.

### Melt–quench protocol

Each system was equilibrated at 50 K and then heated to 2500 K under NPT conditions (Nosé–Hoover thermostat and barostat, zero external pressure) to obtain a homogeneous liquid. After isothermal equilibration at 2500 K, the liquids were quenched to 300 K at nominal cooling rates of 10^11^, 10^13^, and 10^15 ^K/s by linearly rescaling the target temperature while maintaining NPT. The MD time step was 1 fs throughout heating, equilibration, and quenching. Multiple independent simulations (*N* = 5) were conducted for every composition-cooling-rate pair to ensure statistical robustness.

### Spherical nanoindentation setup

Spherical nanoindentation was performed on the quenched glasses. To accommodate the indenter while retaining periodic boundary conditions along *z*, the simulation cell was elongated in *z* and a vacuum layer of 70 Å was inserted above the free surface. Atoms within the bottom 10 Å were fixed to represent a rigid substrate, and all remaining atoms were integrated into the NVE ensemble. A weak thermostat was applied to the mobile region to dissipate plastic heat. A virtual spherical indenter (radius *R* = 30 Å) was driven along +*z* at 0.1 Å/ps (10 m/s). Loading proceeded to a maximum penetration depth of 20 Å, followed by unloading at the same rate.

### Structural analysis: Voronoi free volume and nearest neighbors

All analyzes were performed in OVITO via its Python API^61^. Local atomic polyhedra were characterized using the radical Voronoi construction with metallic radii of 1.28 Å (Cu), 1.43 Å (Al), 1.47 Å (Ti), and 1.60 Å (Zr) serving as effective size parameters. The radical tessellation provides a realistic partitioning of space in dense metallic systems where atomic radii are not strict hard-sphere boundaries. The per-atom excess free space was then defined as

$${V}_{\mathrm{free}}={V}_{\mathrm{Voronoi}}-4/3\pi {R}_{\mathrm{eff}}^{3},$$

where ${V}_{\mathrm{Voronoi}}$ is the atomic Voronoi cell volume and ${R}_{\mathrm{eff}}$ is the species radius used in the radical construction. This metric serves as a relative measure of local packing efficiency, rather than a strict geometric free volume based on non-overlapping spheres.

To assess the validity of this approach, nearest-neighbor (NN) distance distributions were analyzed for all atomic pairs and compared to the nominal contact distances ${R}_{\alpha }+{R}_{\beta }$, confirming that the hard-sphere assumption implicit in the inset-Voronoi definition is not applicable to these metallic glasses.

For each composition, the closest neighbor of every atom were identified using OVITO, and the pairwise NN distances ${d}_{{ij}}$ were classified according to the atomic pair type (*α*–*β*). For each atomic pair type (e.g., Cu–Cu, Cu–Zr, Zr–Zr), histograms of ${d}_{{ij}}$ were constructed with 0.02 Å bin width and normalized to probability density (Supplementary Figs. S22–S24).

### Non-affine deformation and STZ identification

Plastic strain between pre- and post-indentation frames was quantified using the Falk–Langer nonaffine displacement metric ${D}_{\min }^{2}$ with the pre-indentation frame as reference, a 4.0 Å cutoff, and the minimum-image convention^12^. Substrate atoms (*z* < 10 Å) were excluded. STZs were defined from ${D}_{\min }^{2}$ whose threshold is set to *μ*+3*σ* of the far-field distribution for each configuration (atoms at > 2.5*R* from the contact), ensuring rate- and composition-comparable identification of dynamic non-affine excursions (see Supplementary Information S4).

### Load–depth processing and mechanical property extraction

Load-displacement curves were constructed from the indenter output. A 9-point moving average was used to identify ${P}_{\max }$ and ${h}_{\max }$. The initial 20% of the unloading branch was fitted linearly to obtain the contact stiffness ${S}_{0}$.

Hardness was evaluated using both the Oliver–Pharr framework and Meyer’s method^62,63^. For Oliver–Pharr analysis, a spherical indenter with radius *R* = 30 Å, geometry factor $\varepsilon =0.75$, and shape factor *β* = 1.0 was employed. The contact depth and projected contact area were computed as

$${h}_{c}={h}_{\max }-\varepsilon \frac{{P}_{\max }}{{S}_{0}},A=\pi {a}^{2},{a}^{2}=2R{h}_{c}-{h}_{c}^{2}.$$

The hardness and moduli were then obtained as

$$H=\frac{{P}_{\max }}{A},{E}_{r}=\frac{\sqrt{\pi }}{2\beta }\frac{{S}_{0}}{\sqrt{A}},{E}_{s}=\left(1-{\nu }^{2}\right){E}_{r},$$

with $\nu =0.33$ and a rigid indenter assumption.

For Meyer’s method, the projected residual contact area ${A}_{{\rm{Meyer}}}$ was determined from post-indentation surface topography. Pre- and post-indentation surfaces were reconstructed on a regular *x*–*y* grid by averaging the top 5% of atomic *z* coordinates within each bin to define height fields ${z}_{{\rm{pre}}-{\rm{indentation}}}\left(x,y\right)$ and ${z}_{{\rm{post}}-{\rm{indentation}}}\left(x,y\right)$. The residual depth field $\varDelta h\left(x,y\right)={z}_{{\rm{pre}}}\left(x,y\right)-{z}_{{\rm{post}}}\left(x,y\right)$ was computed, and pixels with *Δh* exceeding a statistical threshold $\left(\mu +3\sigma \right)$ were identified as part of the residual imprint. Minor noise was removed by morphological opening and closing. The residual projected area was then evaluated as

$${A}_{\mathrm{Meyer}}={N}_{\mathrm{pix}}\Delta x\Delta y,$$

where ${N}_{{\rm{pix}}}$ is the number of imprint pixels and *ΔxΔy* is the bin area. The corresponding hardness was calculated as

$${H}_{{\rm{Meyer}}}=\frac{{P}_{\max }}{{A}_{{\rm{Meyer}}}}.$$

For spherical tips, the imprint shape was approximately circular, and the equivalent radius ${r}_{{\rm{eq}}}=\sqrt{{A}_{{\rm{Meyer}}}/\pi }$ was also reported. Both ${H}_{{\rm{Oliver}}-{\rm{Pharr}}}$ and ${H}_{{\rm{Meyer}}}$ were evaluated from the same ${P}_{\max }$, enabling a direct comparison between stiffness-based and residual-area-based hardness measurements.

### Serration (“pop-in”) detection

Serrations were identified on smoothed P–h traces via local minima of *dP*/*d*ℎ, with a minimum separation of 0.15 nm to avoid double counting. The serration density *ρ*_pop-in_ is the number of qualified events per nanometer of indenter advance, evaluated over a fixed plastic regime 0.6 ≤ ℎ ≤ 2.0 nm *(h/R* *≥* *0.2*), where contact is fully established, geometric nonlinearity is mild, and burst statistics are stationary across alloys and rates.

### Uncertainty quantification and replicates

All reported quantities are aggregated over *N* = 5 independent seeds per composition-quench rate condition. Figures report mean ± standard deviation unless noted; confidence intervals for regressions/correlations were obtained by nonparametric bootstrap over seeds.

## Supplementary information

Supplementary Information

## Data Availability

Additional raw data generated during the simulations are available upon reasonable request.

## Code availability

All input files, LAMMPS scripts, and Python-based post-processing codes used in this study are publicly available at *GitHub* (https://github.com/bajpaianurag/MetallicGlass_Indentation_QuenchRate_FreeVolume).
