# Supplementary material for: Compositional complexity buffers free-volume sensitivity and serrated flow in metallic glasses
Source: NPJ Comput Mater. 2026 Jan 20;12(1):59. doi: 10.1038/s41524-025-01933-7 (PMC12855008; doi:10.1038/s41524-025-01933-7)
Supplement: Supplementary file 1 — Supplementary Information [file 41524_2025_1933_MOESM1_ESM.pdf]

Supplementary Information

Compositional complexity buffers free-volume sensitivity and serrated flow in metallic glasses

Anurag Bajpai*, Jaemin Wang, Dierk Raabe*

Max Planck Institute for Sustainable Materials, 40237 Düsseldorf, Germany

This PDF file contains:

1. Supplementary Figures: S1 to S24
2. Supplementary Tables: S1 to S5
3. References

S1. Second-nearest-neighbor modified embedded-atom method interatomic potential for Zr-Ti binary systems

The second-nearest-neighbour modified embedded atom method (2NN-MEAM) for a binary alloy is defined by thirteen independent parameters: the cohesive energy E_c, equilibrium nearest-neighbour distance r_e, bulk modulus B, a parameter *d* related to the pressure derivative of the bulk modulus (($\partial B/\partial P$) for the chosen reference structure, the ratio of the elemental electron-density scaling factors $\rho_0^A:\rho_0^B$, and eight screening parameters (four C_{min} and four C_{max}). Details of the 2NN-MEAM formalism are provided in the literature [1-3].

Table S1 – 2NN MEAM potential parameter sets for pure Zr and Ti [4]. The units of cohesive energy E_c, equilibrium nearest-neighbor distance r_e, and bulk modulus B are eV, Å and 10^{12} dyne/cm^2, respectively. The reference structure for Zr and Ti is hcp.

	E_c	r_e	B	A	$\beta^{(0)}$	$\beta^{(1)}$	$\beta^{(2)}$	$\beta^{(3)}$	$t^{(1)}$	$t^{(2)}$	$t^{(3)}$	C_{min}	C_{max}	$d+$	$d-$
Zr	6.36	3.2	0.968	0.68	2.45	1.0	3.0	2.0	6.3	-3.3	-10.0	1.00	1.44	0.00	0.00
Ti	4.87	2.92	1.097	0.66	2.7	1.0	3.0	1.0	6.8	-2.0	-12.0	1.00	1.44	0.00	0.00

Table S2 – 2NN MEAM potential parameter sets for the Zr-Ti binary systems. The units of the cohesive energy E_c, equilibrium nearest-neighbor distance r_e, and bulk modulus B are eV, Å and 10^{12} dyne/cm^2, respectively. "A" represents Zr and "B" represents Ti.

	**Zr-Ti**
Ref. St.	B2-type ZrTi(AB)
E_c	5.585 ($0.5E_c^{Zr}+0.5E_c^{Ti}-0.03$)
r_e	2.93
B	1.73
C_{min} (A-B-A)	0.30
C_{min} (B-A-B)	1.00
C_{min} (A-A-B)	1.35
C_{min} (A-B-B)	1.00
C_{max} (A-B-A)	1.44
C_{max} (B-A-B)	1.44
C_{max} (A-A-B)	2.10
C_{max} (A-B-B)	1.44
$d+$	0.0000 ($0.5d^{Zr}+0.5d^{Ti}$)
$d-$	0.0000 ($0.5d^{Zr}+0.5d^{Ti}$)
$\rho_0^A:\rho_0^B$	1:1

The previously developed unary parameter sets for Zr and Ti, together with the present binary parameters, are summarized in Tables S1 and S2, respectively. The validity of the present potentials was assessed by calculating the fundamental properties of the Zr-Ti systems using the potentials and comparing the calculated properties with ab-initio calculation results and reference experimental data.

First-principles calculations were performed using the Vienna Ab initio Simulation Package (VASP) with the projector augmented-wave (PAW) method and the Perdew–Burke–Ernzerhof (PBE) exchange–correlation functional within the generalized gradient approximation (GGA) [16–20]. A plane-wave cutoff of 520 eV and a Γ-centered 4 × 4 × 4 Monkhorst–Pack mesh were employed. Spin polarization was included. Structural relaxations proceeded in two steps: (*i*) full relaxation of cell volume, shape, and internal coordinates to an energy tolerance of 10^{-4} eV and a force threshold of 0.02 eV/Å; (*ii*) fixed-cell relaxation of internal coordinates with a tighter energy tolerance of 10^{-6} eV. The first-order Methfessel–Paxton smearing (σ = 0.1 eV) was used. All calculations used the "Accurate" precision and the "Fast" electronic minimization algorithm.

Classical calculations were carried out in LAMMPS at 0 K unless noted otherwise, using a radial cutoff of 5 Å. For intermetallic prototypes, supercells of ~1,000 atoms were used; for random solid solutions, cells of 32,000 atoms were employed.

Table S3 – Formation energies (eV/atom) and equilibrium lattice parameters (Å) of representative Zr–Ti intermetallic prototypes from DFT and the present 2NN-MEAM potential.

	Formation Energy (eV/atom)		a (Å)		c (Å)	
	DFT	MD	DFT	MD	DFT	MD
B1_TiZr	0.831409	0.874439	5.5496	5.5628		
B2_TiZr	0.108144	0.03	3.4086	3.3833		
B3_TiZr	2.272984	2.119804	6.1902	5.931		
C14_Ti2Zr	0.11581	0.061568	5.8438	5.4664	7.8393	8.8745
C14_TiZr2	0.435638	0.430919	5.7266	5.7254	9.2453	9.2659
C15_Ti2Zr	0.378165	0.063752	7.7967	7.7197		
C15_TiZr2	0.472537	0.423994	8.1451	8.0827		
C36_Ti2Zr	0.130615	0.062672	5.8468	5.4626	15.5854	17.788
C36_TiZr2	0.452531	0.427558	5.7553	5.7201	18.5149	18.6017
D019_Ti3Zr	0.026535	0.04011	6.033	5.9492	4.7416	4.7913
D019_TiZr3	0.036141	0.073399	6.3028	6.3024	5.0464	4.962
D022_Ti3Zr	0.081776	0.068707	4.2098	4.1403	8.3862	8.5677
D022_TiZr3	0.07602	0.131047	4.3867	4.4117	9.0088	8.817
D03_Ti3Zr	0.126252	0.074623	6.6579	6.6255		
D03_TiZr3	0.121353	0.106993	6.9921	6.9681		
L10_TiZr	0.083062	0.03	3.0705	3.3833	4.2714	3.3833
L12_Ti3Zr	0.086322	0.080559	4.2083	4.1896		
L12_TiZr3	0.079361	0.130714	4.4281	4.4122		

The quality of the Zr–Ti potential was assessed by comparing formation energies at 0 K and lattice parameters for common intermetallic prototypes (B1, B2, B3, $L1_0$, C14, C15, C36, $D0_{19}$, $D0_{22}$, $D0_3$) obtained from molecular simulations with density-functional theory (DFT) results (Table S3). The molecular-simulation values closely track the DFT reference across all structures considered, indicating that the potential reproduces DFT energetics and equilibrium volumes with good fidelity.

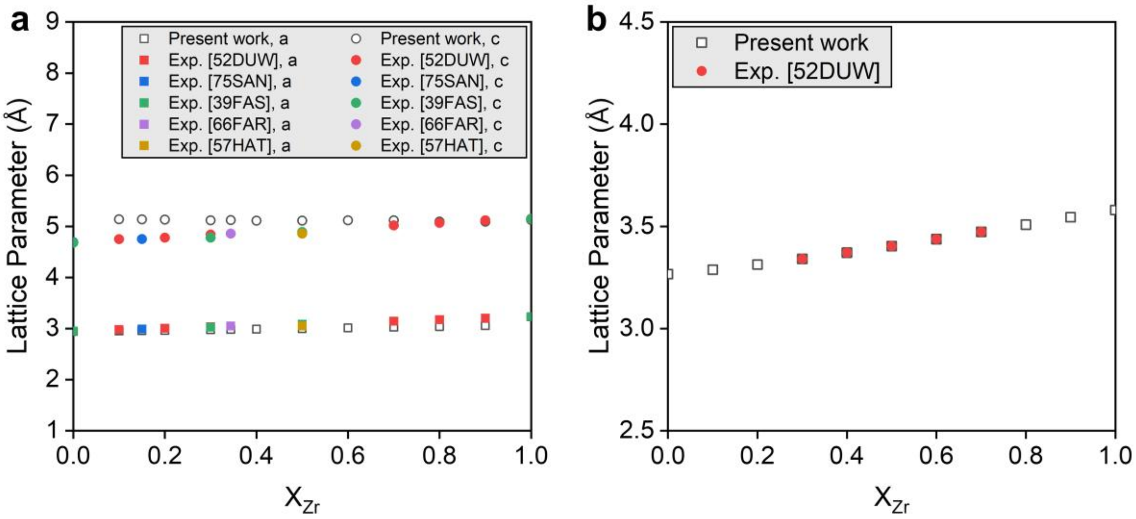

Figure S1 – Lattice parameters of Zr–Ti solid solutions as a function of Zr composition. (a) hcp phase and (b) bcc phase. Experimental values are compared with molecular dynamics results obtained using the present Zr–Ti 2NN MEAM potential.

The experimental Zr–Ti phase diagram shows that the hcp solid solution is stable at low temperatures across the full composition range, whereas the bcc solid solution is stable at elevated temperatures. To further validate the potential for disordered phases, we compared calculated lattice parameters of hcp and bcc random solutions against available experimental data as a function of Zr content (Figure S1) [5-8]. The potential reproduces the experimental trends and magnitudes well; the only notable deviation is a modest overestimation of the c parameter in Ti-rich hcp alloys. Overall, these comparisons support that the developed Zr–Ti 2NN MEAM potential captures the essential structural characteristics of both ordered compounds and random solid solutions across composition.

S2. 2NN MEAM library/parameter files in the LAMMPS format

2NN MEAM library file

```
#elt    lat        z          iel        atwt
#                  alpha                 b0       b1       b2       b3       alat           esub     asub
#                  t0         t1         t2       t3       rhozero  ibar

'Al'    'fcc'  12  1          26.9820
                   4.6855976824          3.200    2.600    6.000    2.600    4.0446507884   3.360    1.160
                   1.00       3.050      0.510    7.750    1.000    3

'Zr'    'hcp'  12  1          91.2200
                   4.4501908328          2.450    1.000    3.000    2.000    3.2000000000   6.360    0.680
                   1.00       6.300      -3.300   -10.00   1.000    3

'Ti'    'hcp'  12  1          47.8800
                   4.7194566335          2.700    1.000    3.000    1.000    2.9200000000   4.870    0.660
                   1.00       6.800      -2.000   -12.00   1.000    3

'Cu'    'fcc'  12  1          63.5460
                   5.1548300830          3.830    2.200    6.000    2.200    3.6133156519   3.540    0.940
                   1.00       2.720      3.040    1.950    1.000    3
```

2NN MEAM parameter file; (1, 2, 3, 4) = (Al, Zr, Ti, Cu)

rc = 5

delr = 0.1

augt1 = 0

erose_form = 2

ialloy = 2

zbl(1,1) = 0

nn2(1,1) = 1

rho0(1) = 1.000

Ec(1,1) = 3.360

re(1,1) = 2.8600

alpha(1,1) = 4.68559768

repuls(1,1) = 0.05

attrac(1,1) = 0.05

Cmin(1,1,1) = 0.49

Cmax(1,1,1) = 2.80

zbl(2,2) = 0

nn2(2,2) = 1

rho0(2) = 1.000

Ec(2,2) = 6.360

re(2,2) = 3.2000

alpha(2,2) = 4.45019083

repuls(2,2) = 0.00

attrac(2,2) = 0.00

Cmin(2,2,2) = 1.00

Cmax(2,2,2) = 1.44

zbl(3,3) = 0

nn2(3,3) = 1

rho0(3) = 1.000

Ec(3,3) = 4.870

re(3,3) = 2.9200

alpha(3,3) = 4.71945663

repuls(3,3) = 0.00

attrac(3,3) = 0.00

Cmin(3,3,3) = 1.00

Cmax(3,3,3) = 1.44

zbl(4,4) = 0

nn2(4,4) = 1

rho0(4) = 1.000

Ec(4,4) = 3.540

re(4,4) = 2.5550

alpha(4,4) = 5.15483008

repuls(4,4) = 0.05

attrac(4,4) = 0.05

Cmin(4,4,4) = 1.21

Cmax(4,4,4) = 2.80

zbl(1,2) = 0

nn2(1,2) = 1

lattce(1,2) = 'b2'

Ec(1,2) = 5.5110

re(1,2) = 2.89000

alpha(1,2) = 4.49280933

repuls(1,2) = 0.0250

attrac(1,2) = 0.0250

Cmin(1,1,2) =0.68

Cmin(2,2,1) =0.68

Cmin(1,2,1) =0.48

Cmin(2,1,1) =0.48

Cmin(1,2,2) =1.08

Cmin(2,1,2) =1.08

Cmax(1,1,2) = 2.80

Cmax(2,2,1) = 2.80

Cmax(1,2,1) = 2.80

Cmax(2,1,1) = 2.80

Cmax(1,2,2) = 1.44

Cmax(2,1,2) = 1.44

zbl(1,3) = 0

nn2(1,3) = 1

lattce(1,3) = 'b2'

Ec(1,3) = 4.3750

re(1,3) = 2.80000

alpha(1,3) = 5.56228620

repuls(1,3) = 0.0250

attrac(1,3) = 0.0250

Cmin(1,1,3) =0.49

Cmin(3,3,1) =1.30

Cmin(1,3,1) =0.722500

Cmin(3,1,1) =0.722500

Cmin(1,3,3) =0.46

Cmin(3,1,3) =0.46

Cmax(1,1,3) = 1.44

Cmax(3,3,1) = 2.80

Cmax(1,3,1) = 2.80

Cmax(3,1,1) = 2.80

Cmax(1,3,3) = 1.44

Cmax(3,1,3) = 1.44

zbl(1,4) = 0

nn2(1,4) = 1

lattce(1,4) = 'b1'

Ec(1,4) = 3.6500

re(1,4) = 2.53000

alpha(1,4) = 4.65645617

repuls(1,4) = 0.0500

attrac(1,4) = 0.0500

Cmin(1,1,4) =0.01

Cmin(4,4,1) =1.21

Cmin(1,4,1) =0.50

Cmin(4,1,1) =0.50

Cmin(1,4,4) =0.95

Cmin(4,1,4) =0.95

Cmax(1,1,4) = 2.80

Cmax(4,4,1) = 2.80

Cmax(1,4,1) = 2.80

Cmax(4,1,1) = 2.80

Cmax(1,4,4) = 2.80

Cmax(4,1,4) = 2.80

zbl(2,3) = 0

nn2(2,3) = 1

lattce(2,3) = 'b2'

Ec(2,3) = 5.5850

re(2,3) = 2.93000

alpha(2,3) = 4.57232984

repuls(2,3) = 0.0000

attrac(2,3) = 0.0000

Cmin(2,2,3) =0.30

Cmin(3,3,2) =1.00

Cmin(2,3,2) =1.35

Cmin(3,2,2) =1.35

Cmin(2,3,3) =1.00

Cmin(3,2,3) =1.00

Cmax(2,2,3) = 1.44

Cmax(3,3,2) = 1.44

Cmax(2,3,2) = 2.10

Cmax(3,2,2) = 2.10

Cmax(2,3,3) = 1.44

Cmax(3,2,3) = 1.44

zbl(2,4) = 0

nn2(2,4) = 1

lattce(2,4) = 'b2'

Ec(2,4) = 5.0700

re(2,4) = 2.82210

alpha(2,4) = 4.78347011

repuls(2,4) = 0.0250

attrac(2,4) = 0.0250

Cmin(2,2,4) =1.00

Cmin(4,4,2) =1.21

Cmin(2,4,2) =1.102500

Cmin(4,2,2) =1.102500

Cmin(2,4,4) =1.102500

Cmin(4,2,4) =1.102500

Cmax(2,2,4) = 1.44

Cmax(4,4,2) = 1.44

Cmax(2,4,2) = 2.80

Cmax(4,2,2) = 2.80

Cmax(2,4,4) = 1.44

Cmax(4,2,4) = 1.44

zbl(3,4) = 0

nn2(3,4) = 1

lattce(3,4) = 'b2'

Ec(3,4) = 4.2650

re(3,4) = 2.75000

alpha(3,4) = 5.19870083

repuls(3,4) = 0.0250

attrac(3,4) = 0.0250

Cmin(3,3,4) =0.49

Cmin(4,4,3) =0.49

Cmin(3,4,3) =1.15

Cmin(4,3,3) =1.15

Cmin(3,4,4) =0.49

Cmin(4,3,4) =0.49

Cmax(3,3,4) = 1.44

Cmax(4,4,3) = 1.44

Cmax(3,4,3) = 2.80

Cmax(4,3,3) = 2.80

Cmax(3,4,4) = 2.80

Cmax(4,3,4) = 2.80

Cmin(1,2,3) =0.74

Cmin(2,1,3) =0.74

Cmin(1,3,2) =0.74

Cmin(3,1,2) =0.74

Cmin(2,3,1) =0.97

Cmin(3,2,1) =0.97

Cmax(1,2,3) = 1.44

Cmax(2,1,3) = 1.44

Cmax(1,3,2) = 1.44

Cmax(3,1,2) = 1.44

Cmax(2,3,1) = 2.80

Cmax(3,2,1) = 2.80

Cmin(1,2,4) =1.01

Cmin(2,1,4) =1.01

Cmin(1,4,2) =1.01

Cmin(4,1,2) =1.01

Cmin(2,4,1) =0.93

Cmin(4,2,1) =0.93

Cmax(1,2,4) = 2.06

Cmax(2,1,4) = 2.06

Cmax(1,4,2) = 2.06

Cmax(4,1,2) = 2.06

Cmax(2,4,1) = 2.80

Cmax(4,2,1) = 2.80

Cmin(1,3,4) =0.68

Cmin(3,1,4) =0.68

Cmin(1,4,3) =0.68

Cmin(4,1,3) =0.68

Cmin(3,4,1) =1.25

Cmin(4,3,1) =1.25

Cmax(1,3,4) = 2.06

Cmax(3,1,4) = 2.06

Cmax(1,4,3) = 2.06

Cmax(4,1,3) = 2.06

Cmax(3,4,1) = 2.80

Cmax(4,3,1) = 2.80

Cmin(2,3,4) =0.72

Cmin(3,2,4) =0.72

Cmin(2,4,3) =1.12

Cmin(4,2,3) =1.12

Cmin(3,4,2) =1.12

Cmin(4,3,2) =1.12

Cmax(2,3,4) = 1.44

Cmax(3,2,4) = 1.44

Cmax(2,4,3) = 2.80

Cmax(4,2,3) = 2.80

Cmax(3,4,2) = 2.80

Cmax(4,3,2) = 2.80

S3. Derivation of Comparative Metrics: QRSI, FVHI, and CCI

Let V_i denote the radical Voronoi volume (Å^3) of atom i in a periodic configuration at 300 K (pre-indentation unless noted). For a system with M atoms,

$$\langle V\rangle = \frac{1}{M}\sum_{i=1}^{M} V_i, \qquad \sigma(V) = \sqrt{\frac{1}{M-1}\sum_{i=1}^{M}(V_i - \langle V\rangle)^2}.$$

Reported 'free volume' is the per-atom radical Voronoi volume V_i (Å^3). When a species-normalized variant is needed, $\tilde{V}_i = V_i/\langle V\rangle_{\alpha(i)}$, where $\alpha(i)$ is the chemical species of atom i.

Voronoi-derived atomic volumes and icosahedral indices follow the convention of Sheng *et al.* [9].

Free-Volume Heterogeneity Index (FVHI)

$$FVHI = \frac{\sigma(V_{free})}{\langle V_{free}\rangle}$$

FVHI quantifies the dispersion of local atomic volumes relative to their mean; it is a scale-free measure of heterogeneity in local packing/dilatation, the structural variable standard Voronoi analyses use to describe free volume. FVHI ≥ 0, with equality only for perfectly uniform packing; larger values indicate broader distributions. FVHI quantifies the dispersion of static free volume at the population level as a surrogate of fictive temperature. It is not used as a local predictor of STZ nucleation. Dynamic dilatancy is assessed *via* serration statistics and D^2_{min} clustering in the main text.

Quench-Rate Sensitivity Index (QRSI)

For a given alloy prepared at two quench rates, "fast" (qf) and "slow" (qs),

$$QRSI = \frac{|FV_{qf} - FV_{qs}|}{FV_{qf}}$$

QRSI is a dimensionless relative change in an intensive free-volume summary between two processing rates; it summarizes the structural susceptibility to quench-rate variation for that alloy [10]. For 3 quench rates, a slope-based variant is used:

$$QRSI = \frac{1}{FV_{\dot{T}_{max}}}\left|\frac{\Delta FV}{\Delta log_{10}\dot{T}}\right|$$

using a finite difference across the extreme rates. $\dot{T}$ is quench rate in K·s^{-1}. We compute FV per seed at each rate; seeds are paired across rates and we form QRSI per pair and report mean ± std.dev.

Configurational Complexity Index (CCI)

The compositional complexity index (CCI) used in this work is defined as the exponential of the ideal configurational entropy of mixing per mole, normalized by the gas constant:

$$CCI \equiv exp\left(\frac{S_{config}}{R}\right) = exp(-\sum_i c_i \ln c_i),$$

where c_i are the atomic fractions with $\sum_i c_i = 1$. This choice is a monotone transform of the thermodynamic quantity that enters the Gibbs free energy $G = H - TS$ for random solutions and is identical to the Hill diversity of order 1 (the "effective number of components"). It therefore admits a direct interpretation: $\mathrm{CCI} = 1$ for a pure element; $\mathrm{CCI} = 2$ for an equiatomic binary; $\mathrm{CCI} = n$ for an equiatomic n-component alloy. Further, it varies continuously with off-equiatomic compositions and microalloying. Mathematically, CCI is permutation-invariant and Schur-concave, increasing with both species richness and evenness, which aligns with the intended notion of "compositional complexity."

In practice, CCI was computed from the nominal alloy stoichiometries with machine-precision evaluation of $c_i \ln(c_i)$ and the convention $0 \ln 0 \equiv 0$ for absent species. Because $\exp(S_{\mathrm{config}}/R)$ is dimensionless and defined on an absolute scale, it provides a single abscissa that increases from binary →ternary →quaternary equiatomic alloys and rises monotonically with small Al additions in the Cu-Zr-Al series, enabling consistent comparison across all compositions examined.

S4. Identification of shear-transformation zones (STZs) from D^2_{min}

For each alloy and quench condition, we computed per-atom D^2_{min} at the maximum indentation depth h_{max} using the pre-indentation configuration as the reference. The local neighborhood was defined by the first minimum of the alloy-specific RDF at 300 K (kept fixed for the entire analysis of that alloy). Frozen atoms (bottom 5 Å) and thermostat shells were excluded from all statistics.

The active (plastic) population was identified by a far-field statistical threshold. We first estimated the spherical contact radius

$$a = \sqrt{2Rh_{max} - h^2_{max}},$$

with indenter radius R=30 Å and h_{max}=20 Å. A far-field region was defined as atoms with lateral distance $r = \sqrt{(x - x_0)^2 - (y - y_0)^2} > 3a$ from the contact point (x_0, y_0). The far-field D^2_{min} values (elastic background) were used to set a threshold

$$D^2_{thr} = \mu_{far} + 3\sigma_{far},$$

where μ_{far} and σ_{far} are the mean and standard deviation of far-field D^2_{min}.

Atoms with $D^2_{min} \geq D^2_{thr}$ were voxelized on a 2.5 Å cubic grid (≈ nearest-neighbor spacing) and 26-connected components were labeled to obtain physically contiguous events. Clusters smaller than 10 voxels were discarded as noise. For each configuration, we calculate STZ count**,** total plastic-zone volume, largest-STZ volume, and equivalent plastic-zone radius $R_{eq} = (3V_{tot}/4\pi)^{1/3}$.

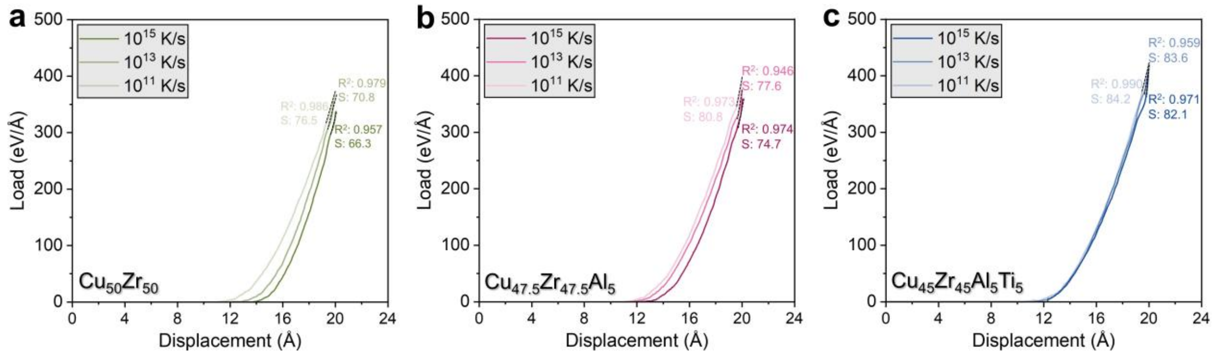

Figure S2 – Unloading fits and contact stiffness from Oliver–Pharr analysis. Representative nanoindentation load-displacement curves for (a) $Cu_{50}Zr_{50}$, (b) $Cu_{47.5}Zr_{47.5}Al_5$, and (c) $Cu_{45}Zr_{45}Al_5Ti_5$ at quench rates 10^{11}, 10^{13} and 10^{15} K·s^{-1}. The unloading branch is fitted to $P(h) = A(h - h_f)^m$ over the upper unloading window; the contact stiffness is obtained as $S = \mathrm{d}P/\mathrm{d}h|_{h\max} = mA(h_{\max} - h_f)^{m-1}$ (black dashed segment, values annotated next to each curve, units: eV·Å^{-2}). Goodness-of-fit (R^2) is shown alongside S. A systematic trend is evident: at fixed alloy, faster quench (higher free volume) yields lower S; at fixed quench rate, higher compositional complexity (binary → ternary → quaternary) increases S, consistent with the modulus trends reported in the main text. Note that all the indents were done to a fixed depth of 20 Å.

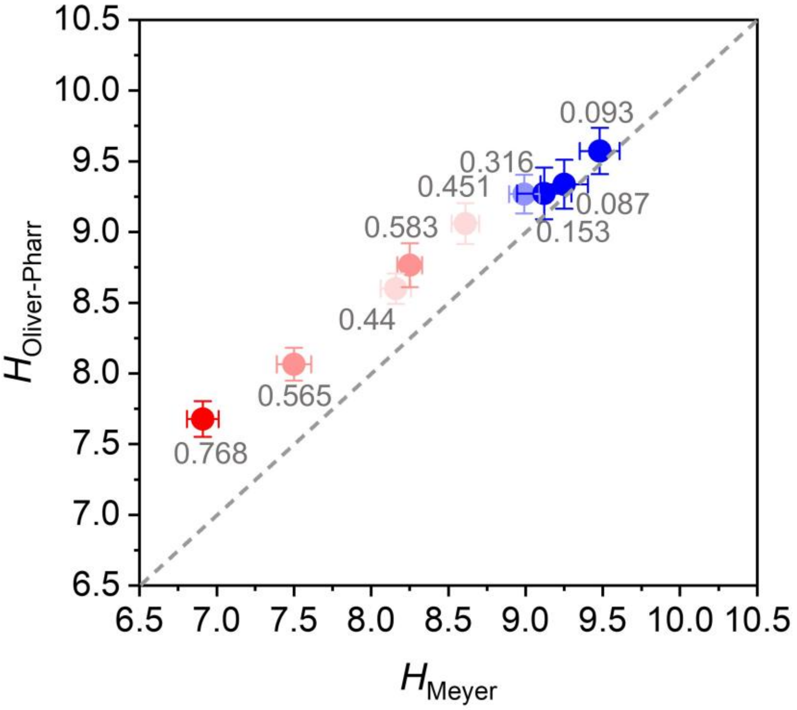

Figure S3 – Comparison of indentation hardness by the Oliver–Pharr method $H_{Oliver\text{-}Pharr}$ and Meyer hardness H_{Meyer} for the studied alloys (GPa). Each marker shows the mean with horizontal/vertical error bars (± s.d.). The dashed line is the 1:1 locus $H_{Oliver\text{-}Pharr} = H_{Meyer}$. Marker color encodes the magnitude of the difference $\Delta H = H_{Oliver\text{-}Pharr} - H_{Meyer}$ (red = larger $|\Delta H|$, blue = smaller $|\Delta H|$), and the number next to each point reports ΔH for that alloy.

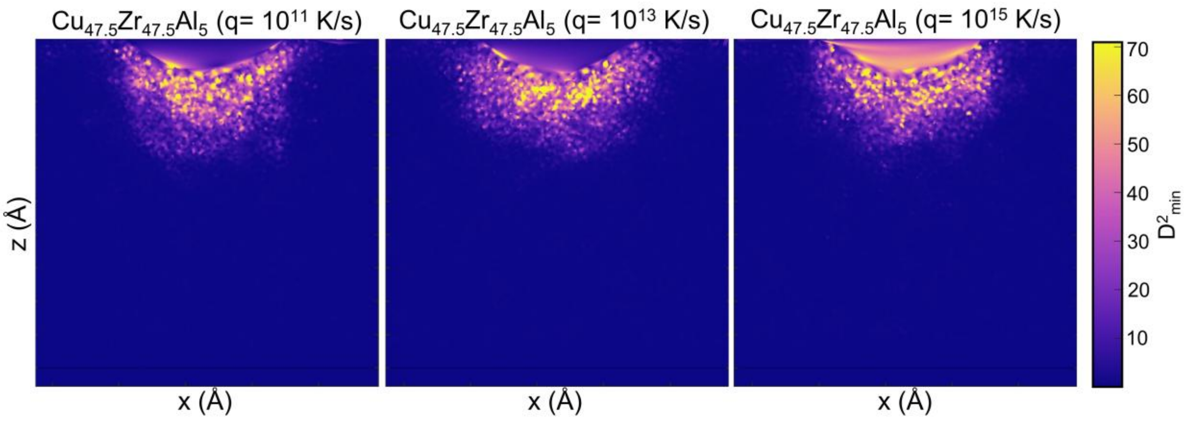

Figure S4 – Deformation mechanisms under nanoindentation. Non-affine displacement (D^2_{min}) fields at maximum depth for $Cu_{47.5}Zr_{47.5}Al_5$ MG, for various quench rates.

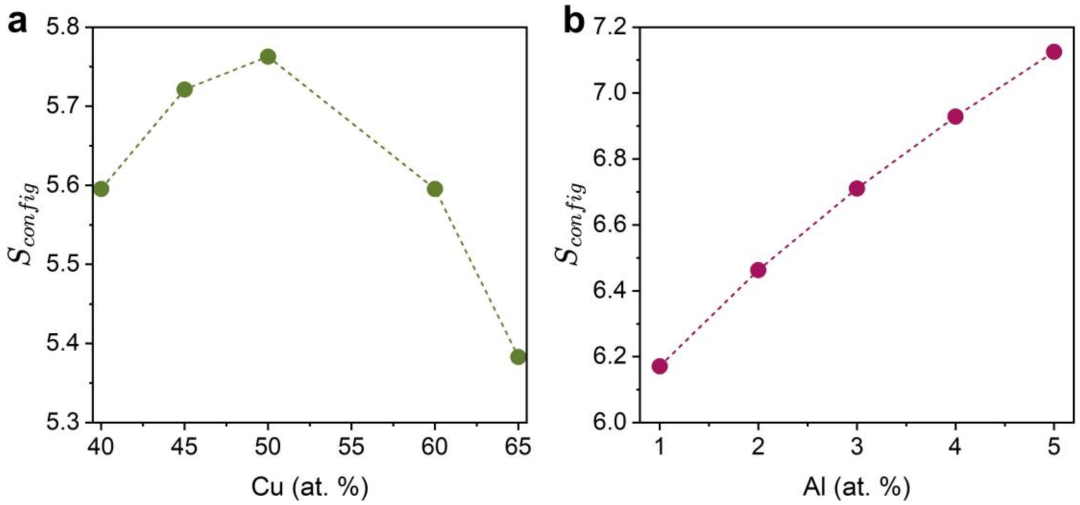

Figure S5 – $S_{\mathrm{config}} = -\mathrm{R}\sum x_i \ln x_i$ for **(a)** the binary sweep Cu_xZr_{100-x} (values plotted in units of R; $\mathrm{Jmol^{-1}K^{-1}}$). The entropy peaks near equiatomic $Cu_{50}Zr_{50}$ and decreases toward Cu-rich or Zr-rich limits, reflecting maximal compositional disorder at $x \approx 50$; **(b)** the Al-microalloyed series $Cu_{50-z/2}Zr_{50-z/2}Al_z$ with $z = 1\ldots5$ at.% (again plotted in units of R). These trends provide the quantitative basis for the compositional-complexity descriptor used in the main text.

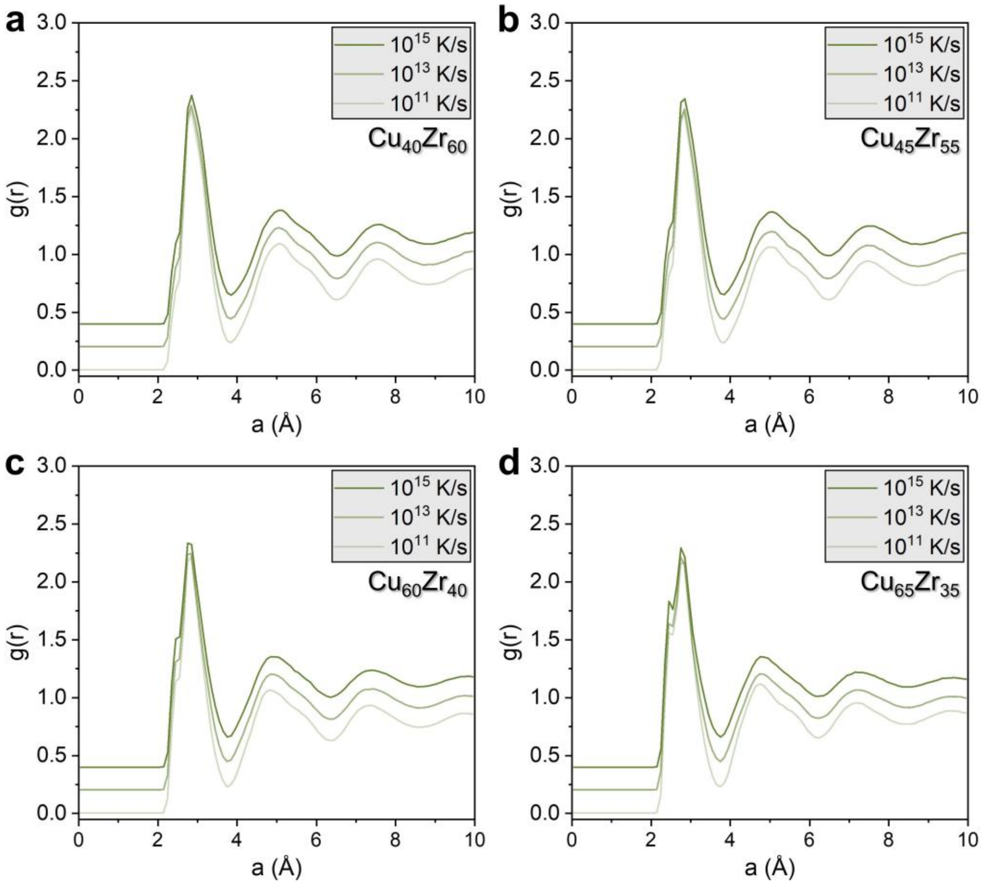

Figure S6 – Total radial distribution functions, $g(r)$, for $Cu_{40}Zr_{60}$ **(a)**, $Cu_{45}Zr_{55}$ **(b)**, $Cu_{60}Zr_{40}$ **(c)**, and $Cu_{65}Zr_{35}$ **(d)** vitrified at 10^{11}, 10^{13} and 10^{15} Ks^{-1}. For each composition, decreasing quench rate sharpens and raises the first peak and deepens the first minimum, while the second-peak split becomes more discernible, signatures of enhanced short- and medium-range order. The magnitude of these rate-induced changes grows as the composition moves away from equiatomic composition.

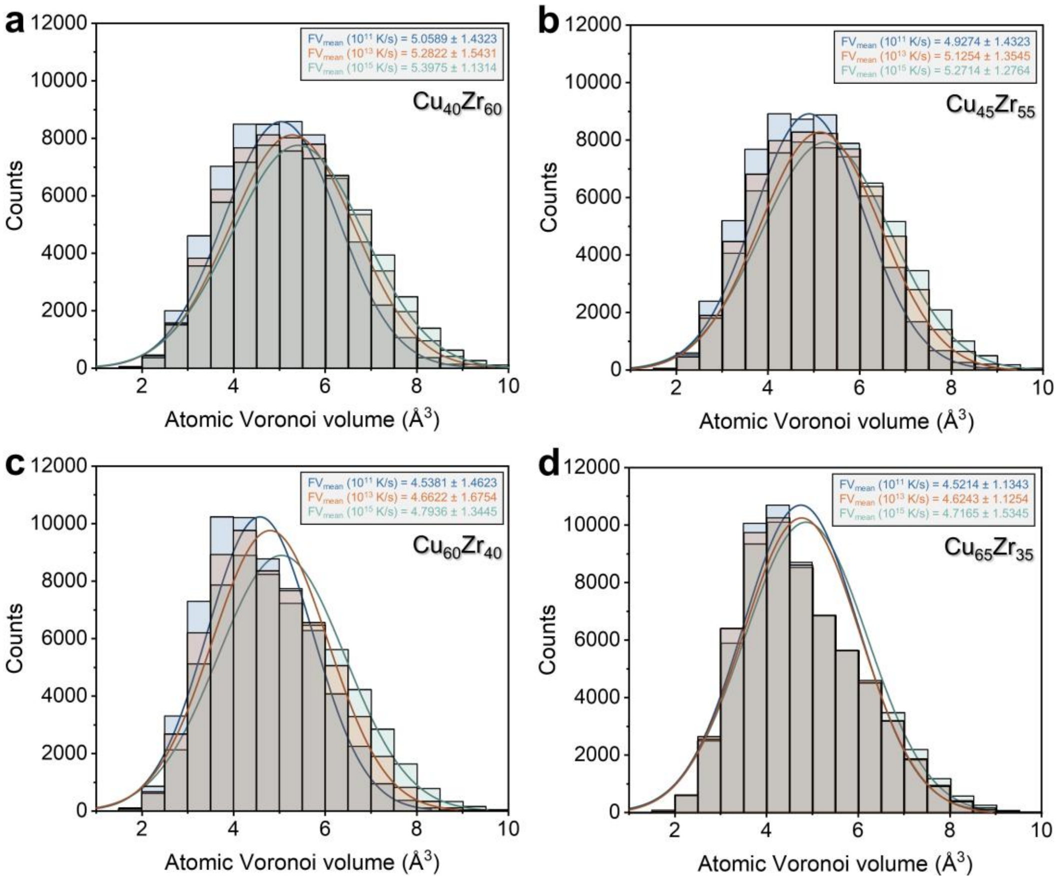

Figure S7 – Distributions of atomic Voronoi volumes for **(a)** $Cu_{40}Zr_{60}$, **(b)** $Cu_{45}Zr_{55}$, **(c)** $Cu_{60}Zr_{40}$ and **(d)** $Cu_{65}Zr_{35}$ quenched at 10^{11}, 10^{13}, and 10^{15} K·s^{-1}. Volumes are obtained from radical Voronoi tessellation, showing progressive narrowing and reduced rate-sensitivity as we move near to equiatomic composition. Histograms (bins fixed across panels) are overlaid with kernel-density estimates. Faster quench broadens the distributions and shifts the mean to larger volumes.

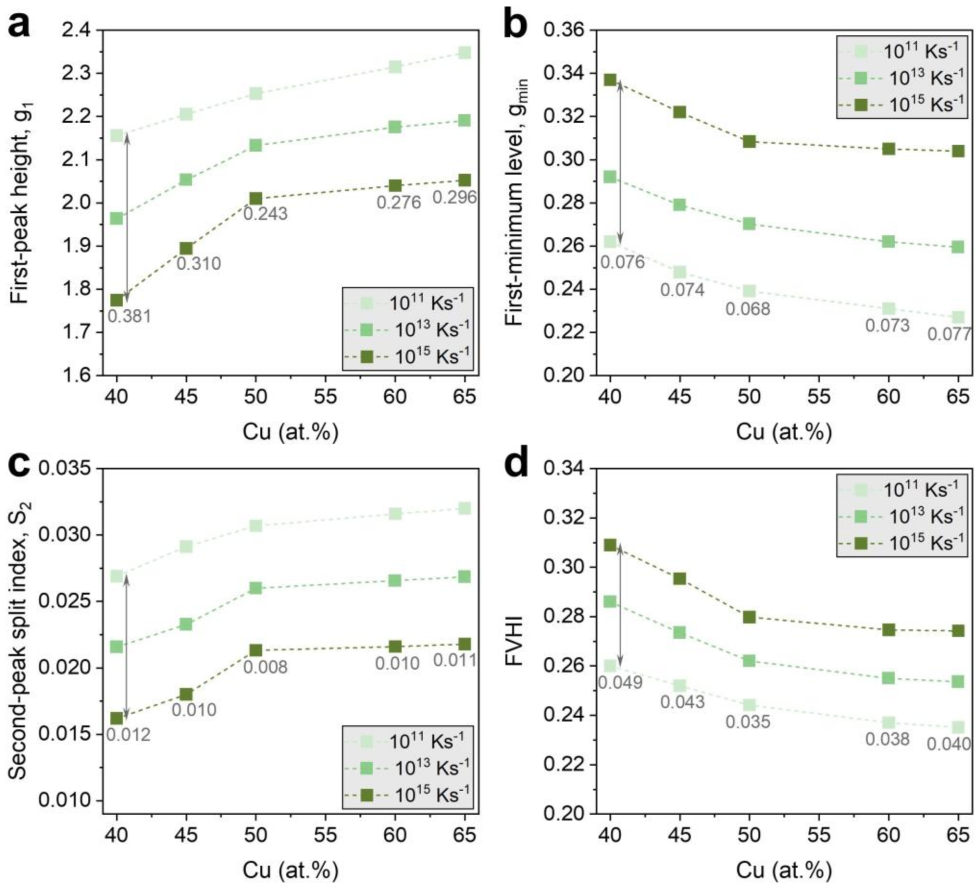

Figure S8 – Composition and quench-rate dependent structural metrics in Cu_xZr_{100-x} metallic glasses sweep. (a) First-peak height of the pair distribution function, g_1; **(b)** first-minimum level, g_{min}; **(c)** second-peak split index, S_2 (a measure of medium-range order); and **(d)** free-volume heterogeneity index (FVHI). Cu content varies from 40 to 65 at.% (balance Zr). Three cooling rates are compared: 10^{11}, 10^{13} and 10^{15} Ks^{-1} (square markers; dashed lines are guides to the eye). Increasing Cu content raises g_1 and S_2 while lowering g_{min}, consistent with enhanced short- and medium-range ordering; faster quenching amplifies these trends. FVHI decreases with Cu enrichment and increases with quench rate, indicating greater free-volume heterogeneity in more rapidly quenched metallic glasses.

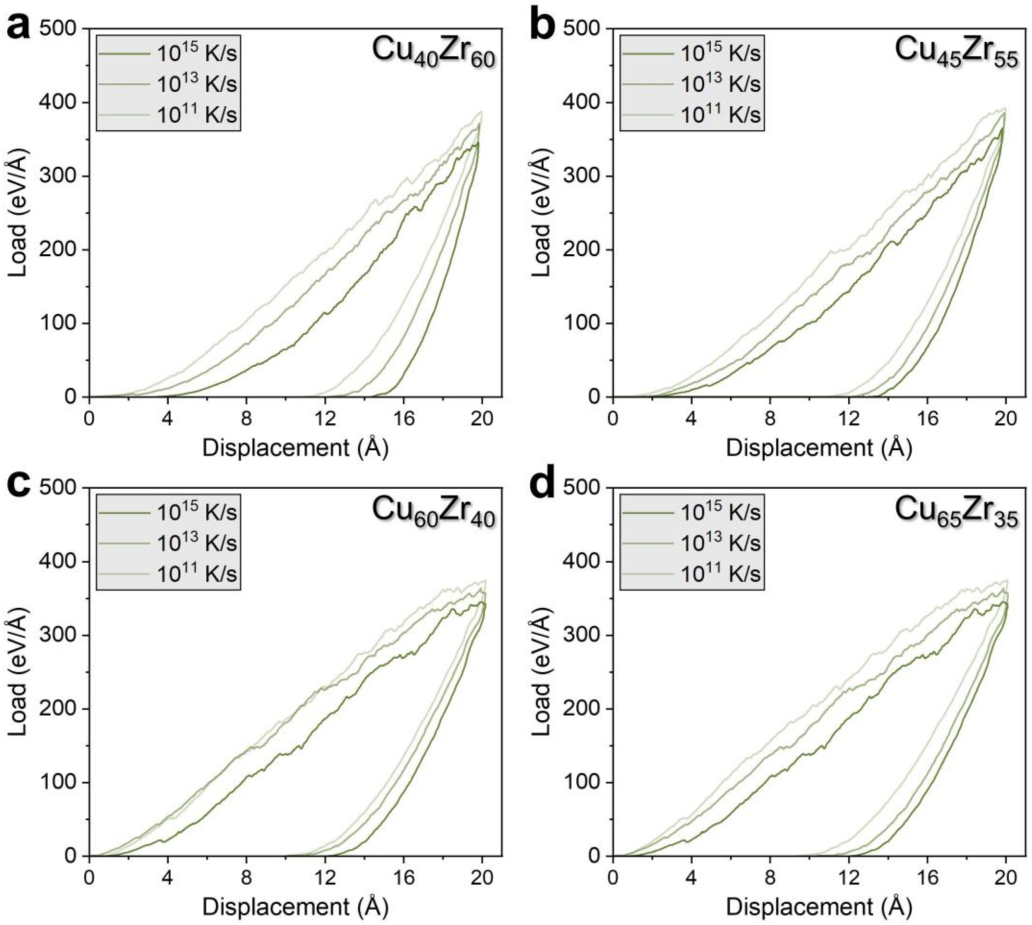

Figure S9 – Quench rate dependence of load-depth curves for **(a)** $Cu_{40}Zr_{60}$, **(b)** $Cu_{45}Zr_{55}$, **(c)** $Cu_{60}Zr_{40}$ and **(d)** $Cu_{65}Zr_{35}$, showing reduced sensitivity to load and depth as composition moves closer to equiatomic $Cu_{50}Zr_{50}$.

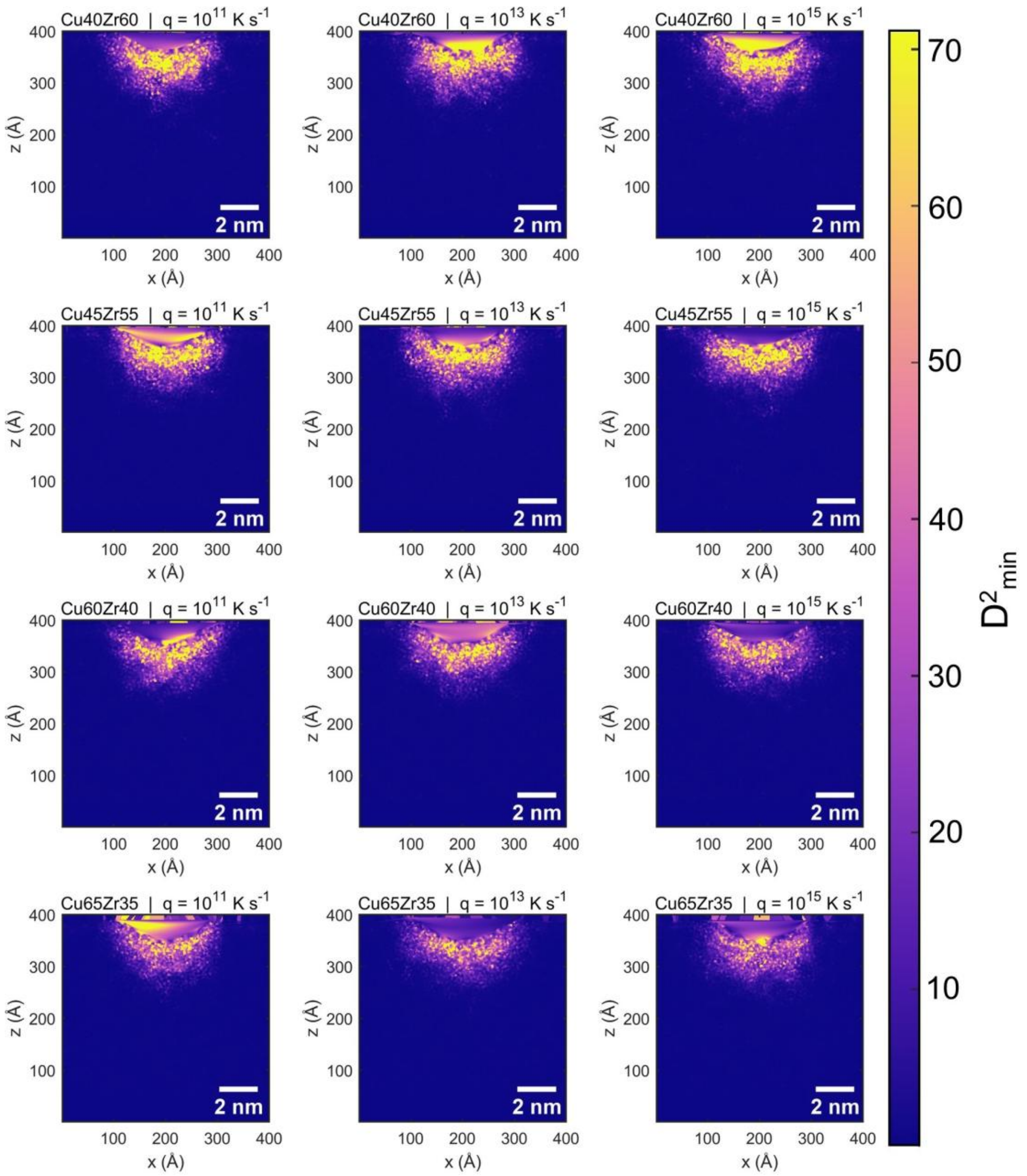

Figure S10 – Deformation mechanisms for Cu-Zr sweep under nanoindentation. Non-affine displacement (D^2_{min}) fields at maximum depth for Cu_xZr_{100-x} (x = 40, 45, 60, 65 at.%) MGs, for various quench rates.

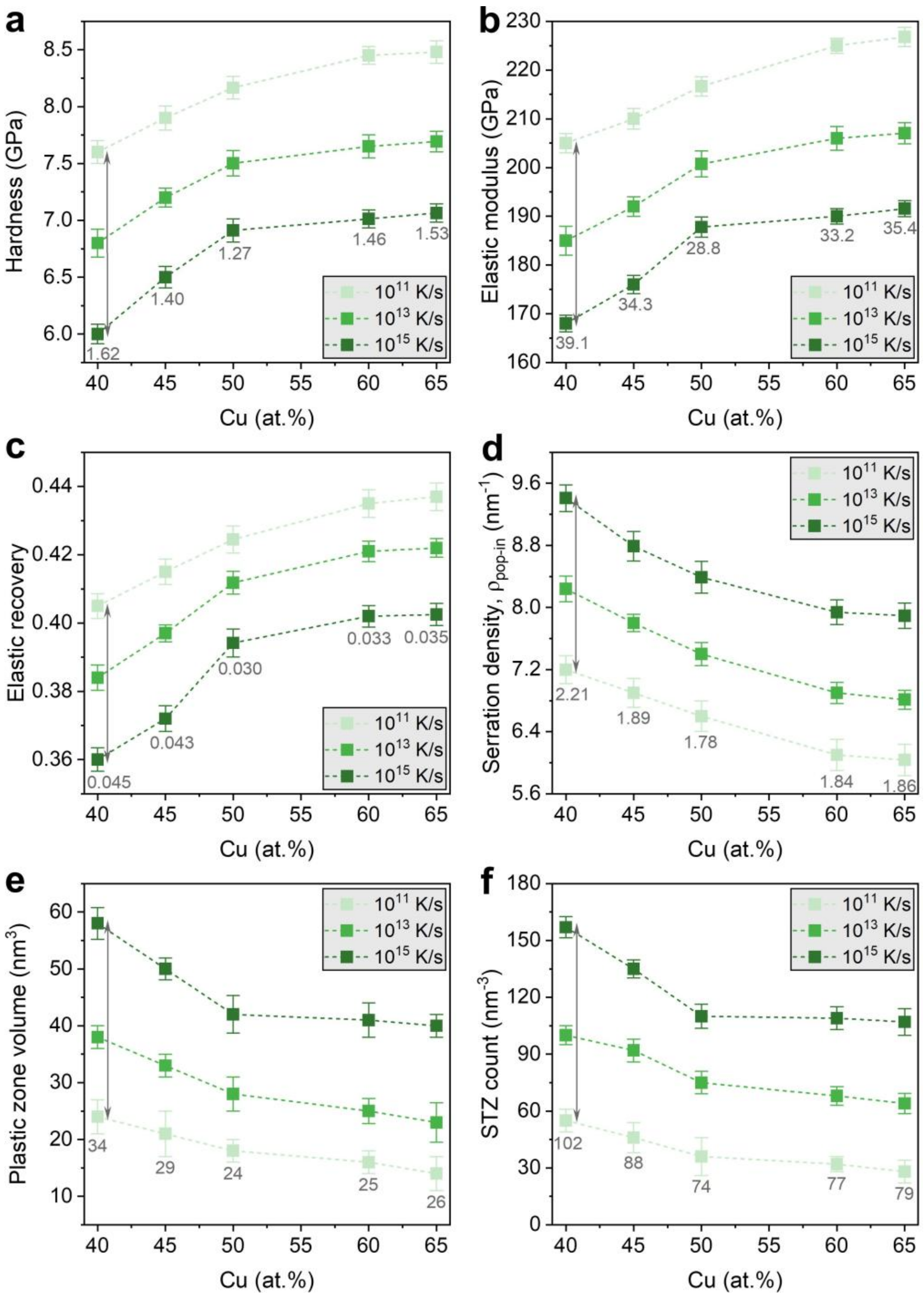

Figure S11 – Composition and quench-rate dependent mechanical response of Cu–Zr glasses from instrumented nanoindentation. **(a)** Hardness, **(b)** elastic modulus, **(c)** elastic recovery, **(d)** serration (pop-in) density $\rho_{\text{pop-in}}$, **(e)** plastic-zone volume, and **(f)** shear-transformation-zone (STZ) count, plotted versus Cu content (40-65 at.%). Three cooling rates are compared: 10^{11}, 10^{13} and 10^{15} Ks^{-1} (square markers; dashed lines are guides). At fixed composition, slower cooling systematically yields higher hardness, modulus, and elastic recovery, whereas faster quenching increases $\rho_{\text{pop-in}}$, plastic-zone volume, and STZ density, consistent with larger frozen-in free volume. Increasing Cu content strengthens and stiffens the metallic glass (rising hardness/modulus and elastic recovery) while suppressing serration activity and reducing plastic-zone volume and STZ density, indicating improved packing and resistance to shear localization. Error bars denote standard deviations; all measurements are evaluated at 300 K on relaxed structures.

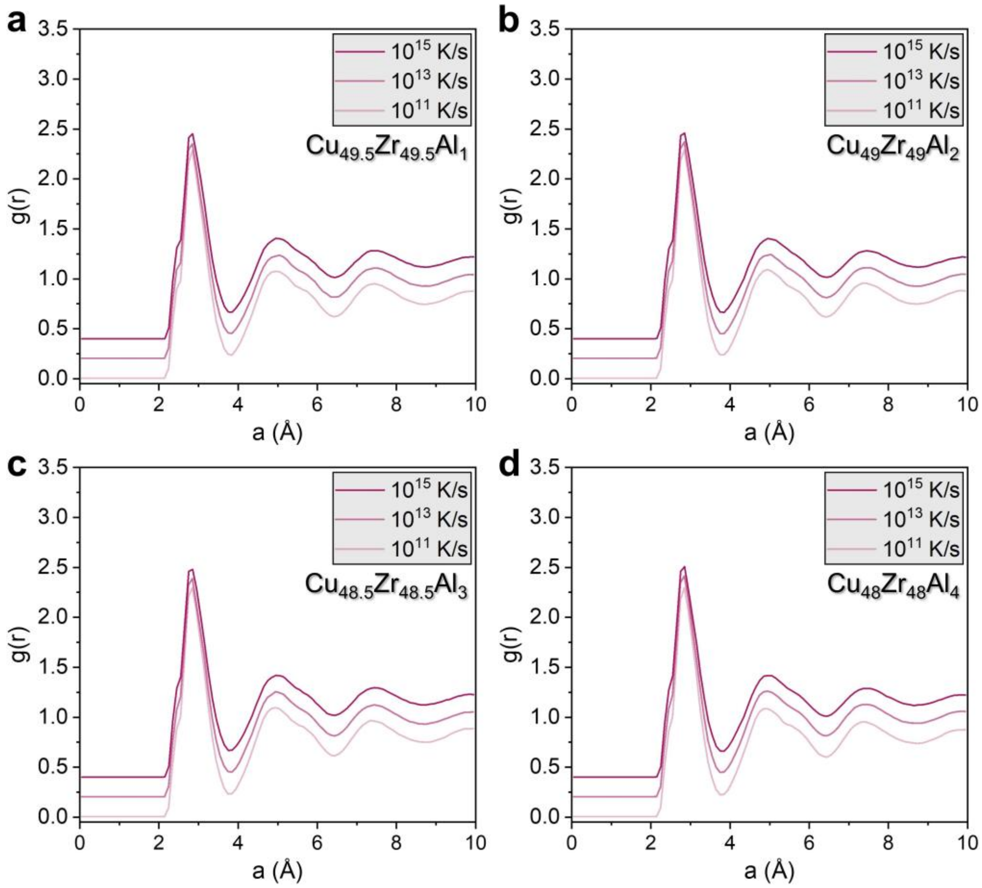

Figure S12 – Total radial distribution functions, g(r), for the $Cu_{50-z/2}Zr_{50-z/2}Al_z$ series at z=1 **(a)**, z=2 **(b)**, z=3 **(c)**, and z=4 **(d)**, vitrified at 10^{11}, 10^{13} and 10^{15} Ks^{-1}. Decreasing quench rate sharpens/raises the first peak and deepens the first minimum in every alloy, with a clearer second-peak split at slower rates, indicating enhanced short- and medium-range order.

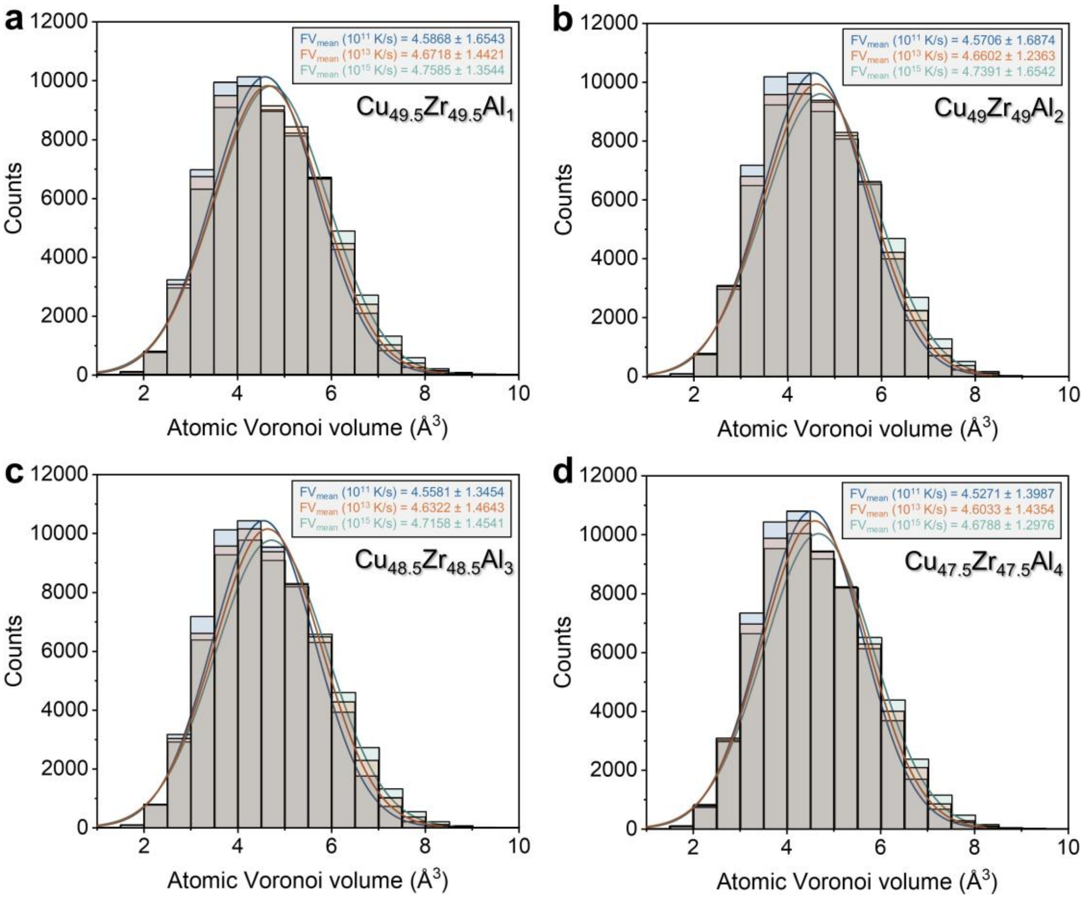

Figure S13 – Distributions of atomic Voronoi volumes for **(a)** $Cu_{49.5}Zr_{49.5}Al_1$, **(b)** $Cu_{49}Zr_{49}Al_2$, **(c)** $Cu_{48.5}Zr_{48.5}Al_3$ and **(d)** $Cu_{48}Zr_{48}Al_4$ quenched at 10^{11}, 10^{13}, and 10^{15} K·s^{-1}. Volumes are obtained from radical Voronoi tessellation, showing progressive narrowing and reduced rate-sensitivity as we move near towards higher compositional complexity. Histograms (bins fixed across panels) are overlaid with kernel-density estimates. Faster quench broadens the distributions and shifts the mean to larger volumes.

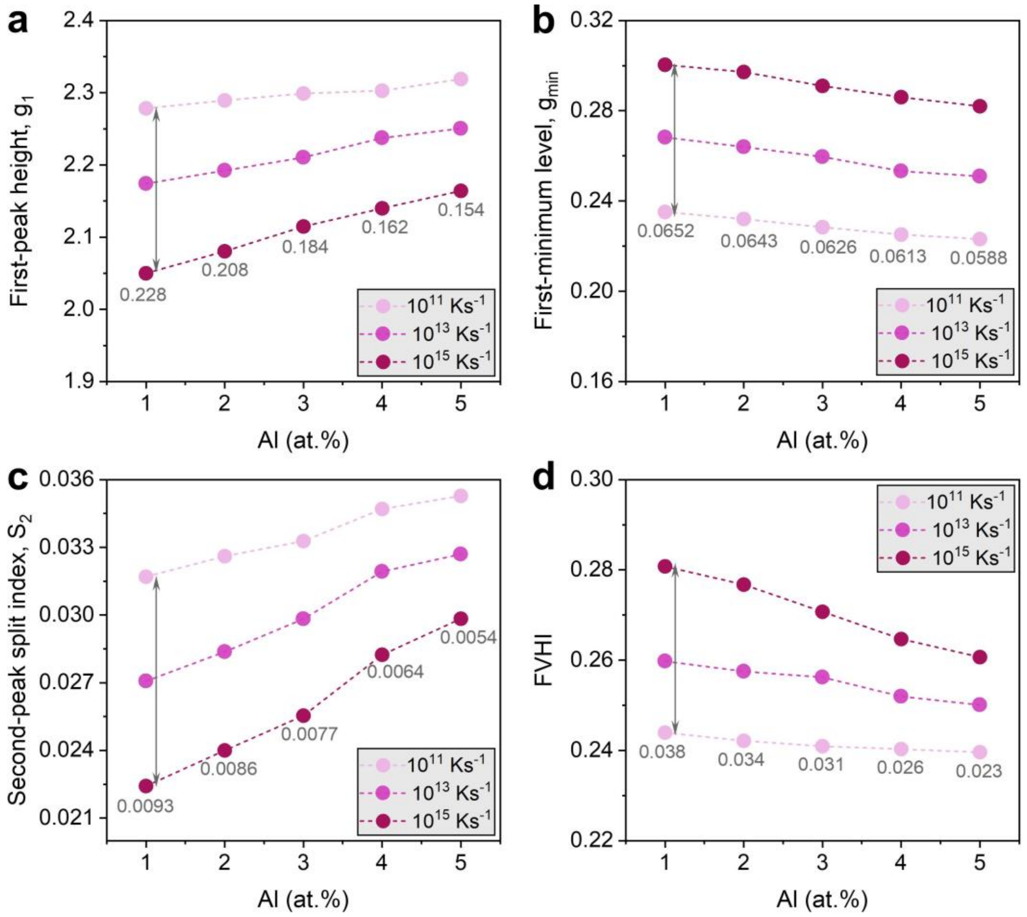

Figure S14 – Composition and quench-rate dependent structural metrics in $Cu_{50-z/2}Zr_{50-z/2}Al_z$ metallic glasses microalloying series. **(a)** First-peak height of the pair distribution function, g_1; **(b)** first-minimum level, g_{min}; **(c)** second-peak split index, S_2 (a measure of medium-range order); and **(d)** free-volume heterogeneity index (FVHI). Al content varies from 1 to 5 at.%. Three cooling rates are compared: 10^{11}, 10^{13} and 10^{15} Ks^{-1} (circle markers; dashed lines are guides to the eye). Increasing Al content raises g_1 and S_2 while lowering g_{min}, consistent with enhanced short- and medium-range ordering; faster quenching amplifies these trends. FVHI decreases with Al enrichment and increases with quench rate, indicating greater free-volume heterogeneity in more rapidly quenched metallic glasses.

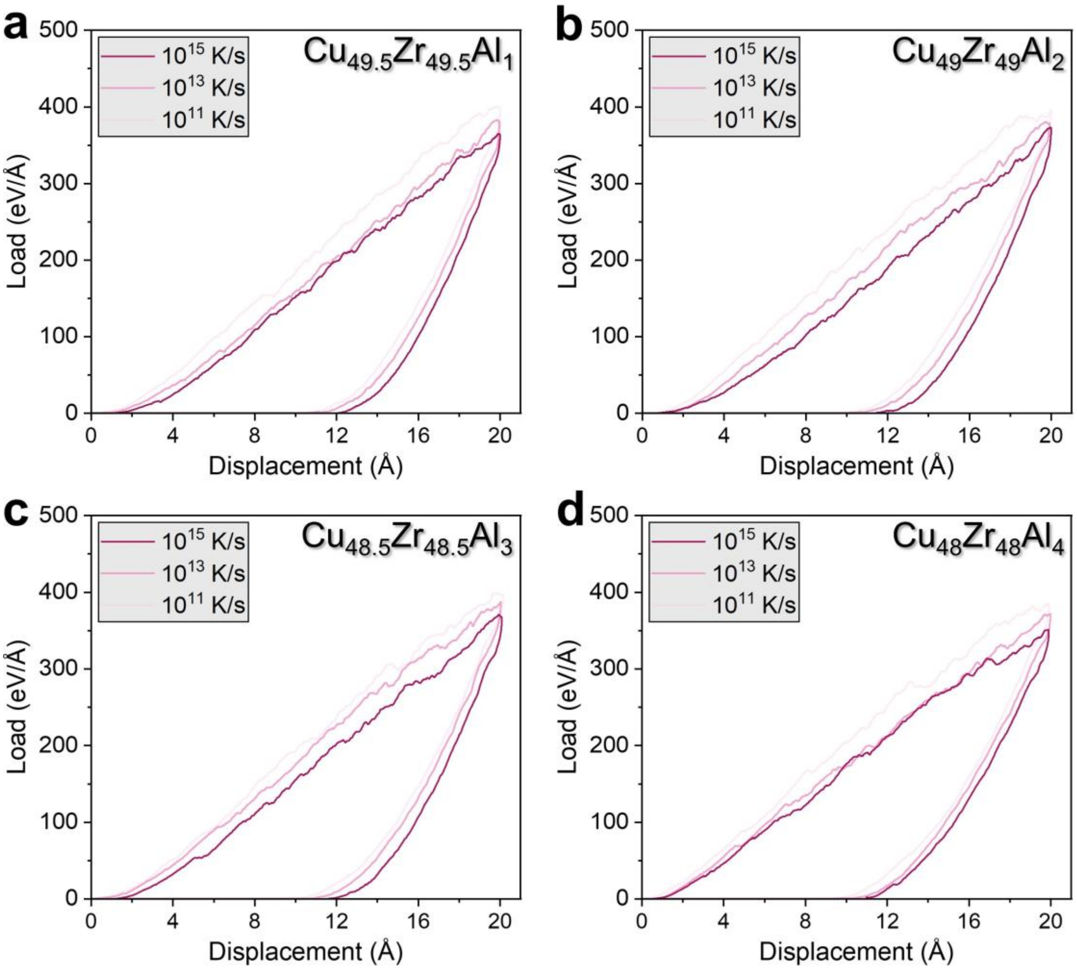

Figure S15 – Quench rate dependence of load-depth curves for **(a)** $Cu_{49.5}Zr_{49.5}Al_1$, **(b)** $Cu_{49}Zr_{49}Al_2$, **(c)** $Cu_{48.5}Zr_{48.5}Al_3$ and **(d)** $Cu_{48}Zr_{48}Al_4$, showing reduced serration and higher load with increasing Al content and slower cooling.

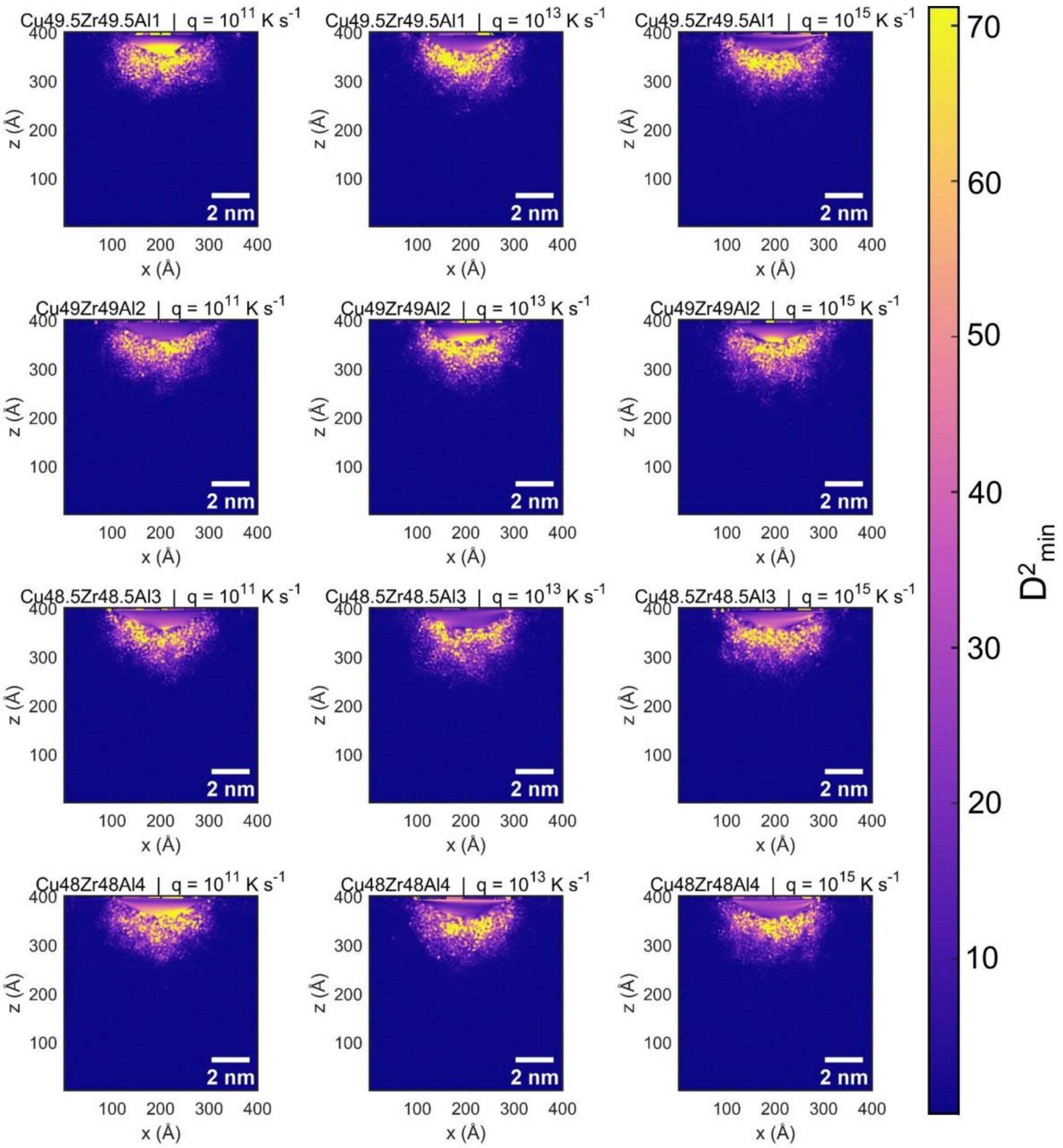

Figure S16 – Deformation mechanisms for Cu-Zr-Al microalloying series under nanoindentation. Non-affine displacement (D^2_{min}) fields at maximum depth for $Cu_{50-z/2}Zr_{50-z/2}Al_z$ (z = 1-4 at.%) MGs, for various quench rates.

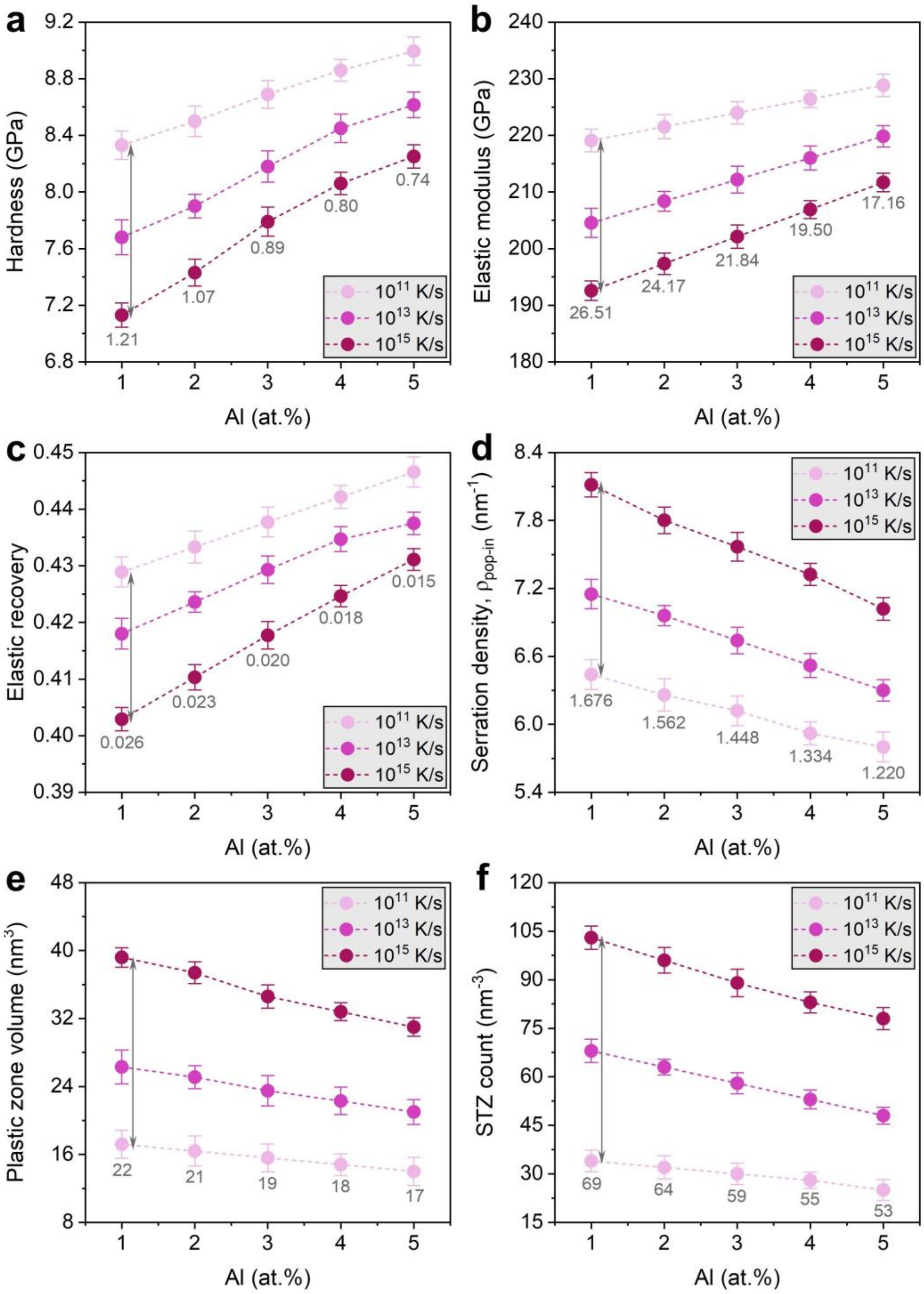

Figure S17 – Composition and quench-rate dependent mechanical response of $Cu_{50-z/2}Zr_{50-z/2}Al_z$ metallic glasses microalloying series from instrumented nanoindentation. (a) Hardness, **(b)** elastic modulus, **(c)** elastic recovery, **(d)** serration (pop-in) density $\rho_{pop\text{-}in}$, **(e)** plastic-zone volume, and **(f)** shear-transformation-zone (STZ) count, plotted versus Al content (1-5 at.%). Three cooling rates are compared: 10^{11}, 10^{13} and 10^{15} Ks^{-1} (circle markers; dashed lines are guides). At fixed composition, slower cooling systematically yields higher hardness, modulus, and elastic recovery, whereas faster quenching increases $\rho_{pop\text{-}in}$, plastic-zone volume, and STZ density, consistent with larger frozen-in free volume. Increasing Al content strengthens and stiffens the metallic glass (rising hardness/modulus and elastic recovery) while suppressing serration activity and reducing plastic-zone volume and STZ density, indicating improved packing and resistance to shear localization. Error bars denote standard deviations; all measurements are evaluated at 300 K on relaxed structures.

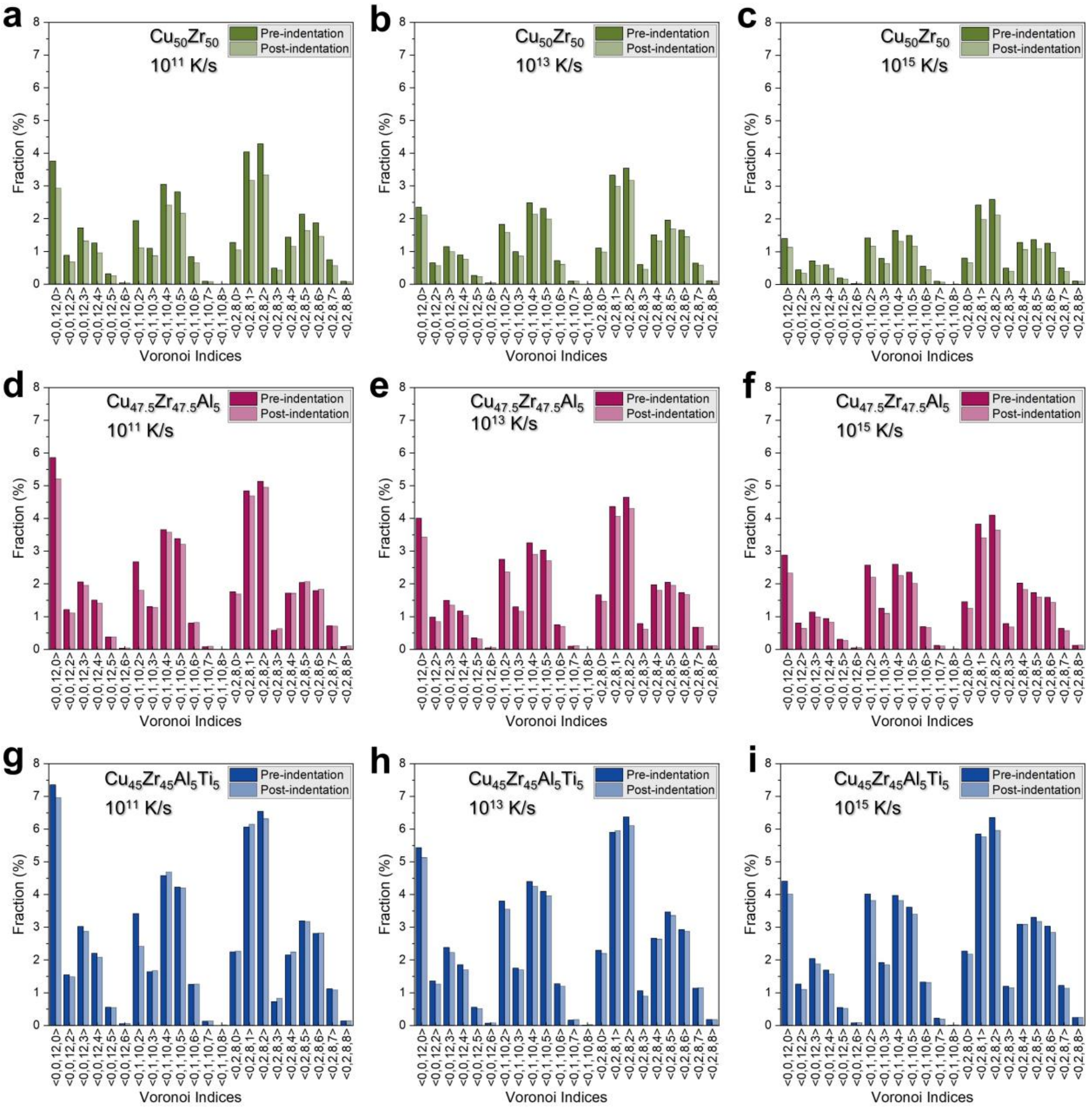

Figure S18 – Voronoi motif populations before and after indentation across alloys and quench rates. (a–c) $Cu_{50}Zr_{50}$, (d–f) $Cu_{47.5}Zr_{47.5}Al_5$, and (g–i) $Cu_{45}Zr_{45}Al_5Ti_5$ at quench rates 10^{11}, 10^{13}, and 10^{15} $K \cdot s^{-1}$, respectively. Bars give the fraction of atoms (%) assigned to each listed Voronoi index; darker bars are for the pre-indentation state; lightly shaded bars are for the post-indentation state (after unloading). For each panel, the sum over indices equals the reported icosahedral-family fraction for that alloy/rate in Figure 5 of the main manuscript.

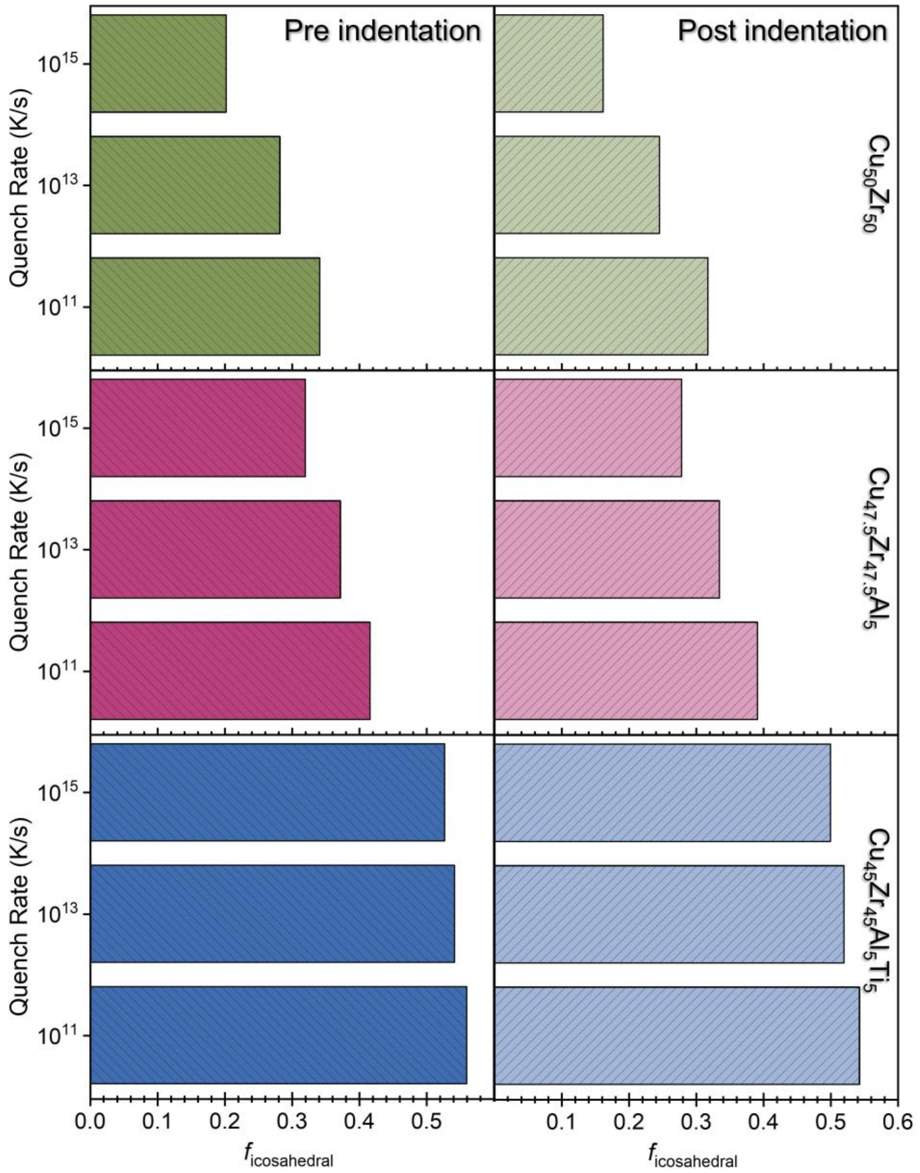

Figure S19 – Fraction of icosahedral motifs $f_{\text{icosahedral}}$ before and after indentation for $Cu_{50}Zr_{50}$, $Cu_{47.5}Zr_{47.5}Al_5$, and $Cu_{45}Zr_{45}Al_5Ti_5$ MGs and quench rate (10^{11}, 10^{13}, 10^{15} $K{\cdot}s^{-1}$); More complex alloys start with higher $f_{\text{icosahedral}}$ and suffer a smaller post-indentation loss, consistent with greater packing stability.

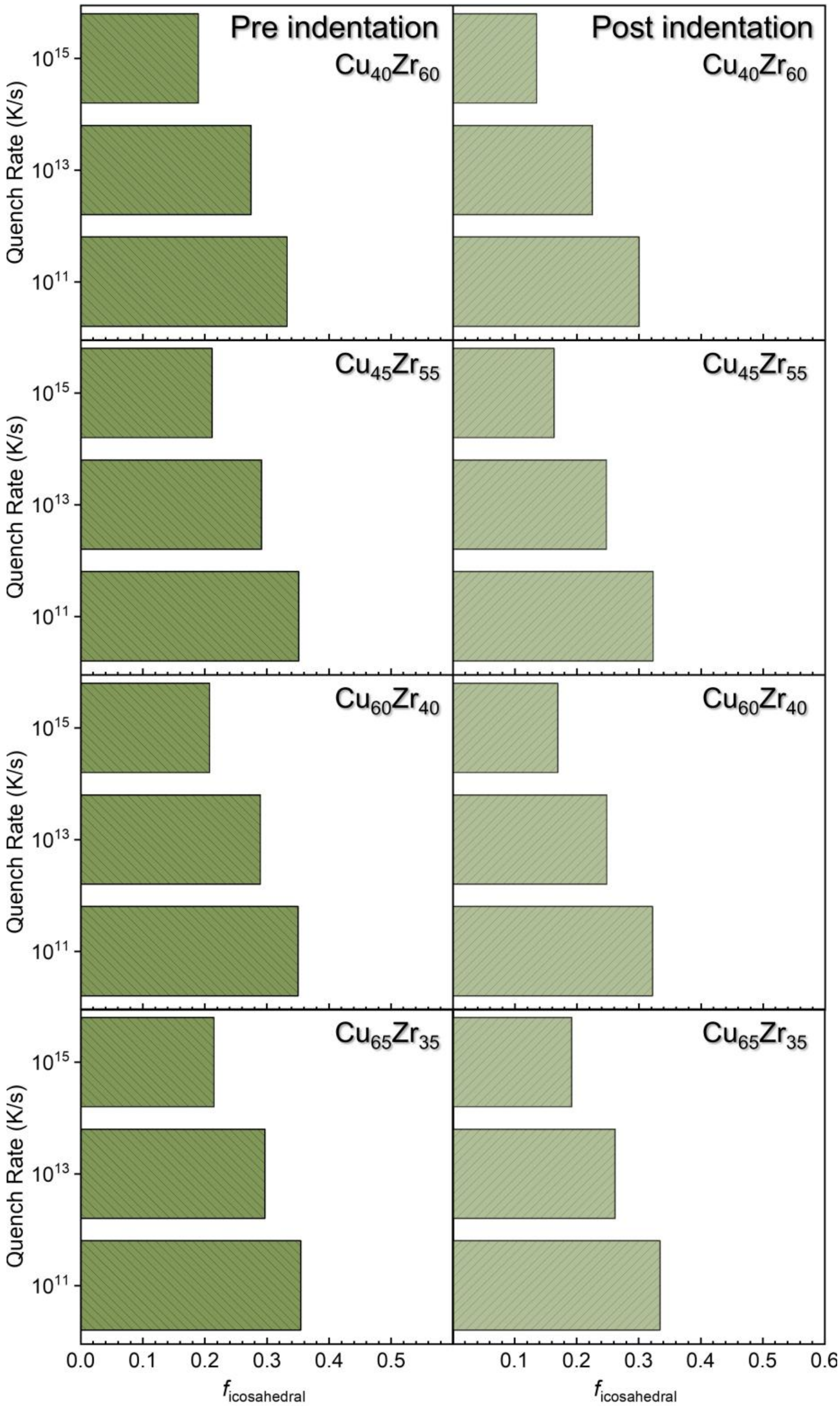

Figure S20 – Fraction of icosahedral motifs $f_{\text{icosahedral}}$ before and after indentation for Cu_xZr_{100-x} (x = 40, 45, 60, 65 at.%) composition sweep at different quench rates (10^{11}, 10^{13}, 10^{15} $K{\cdot}s^{-1}$).

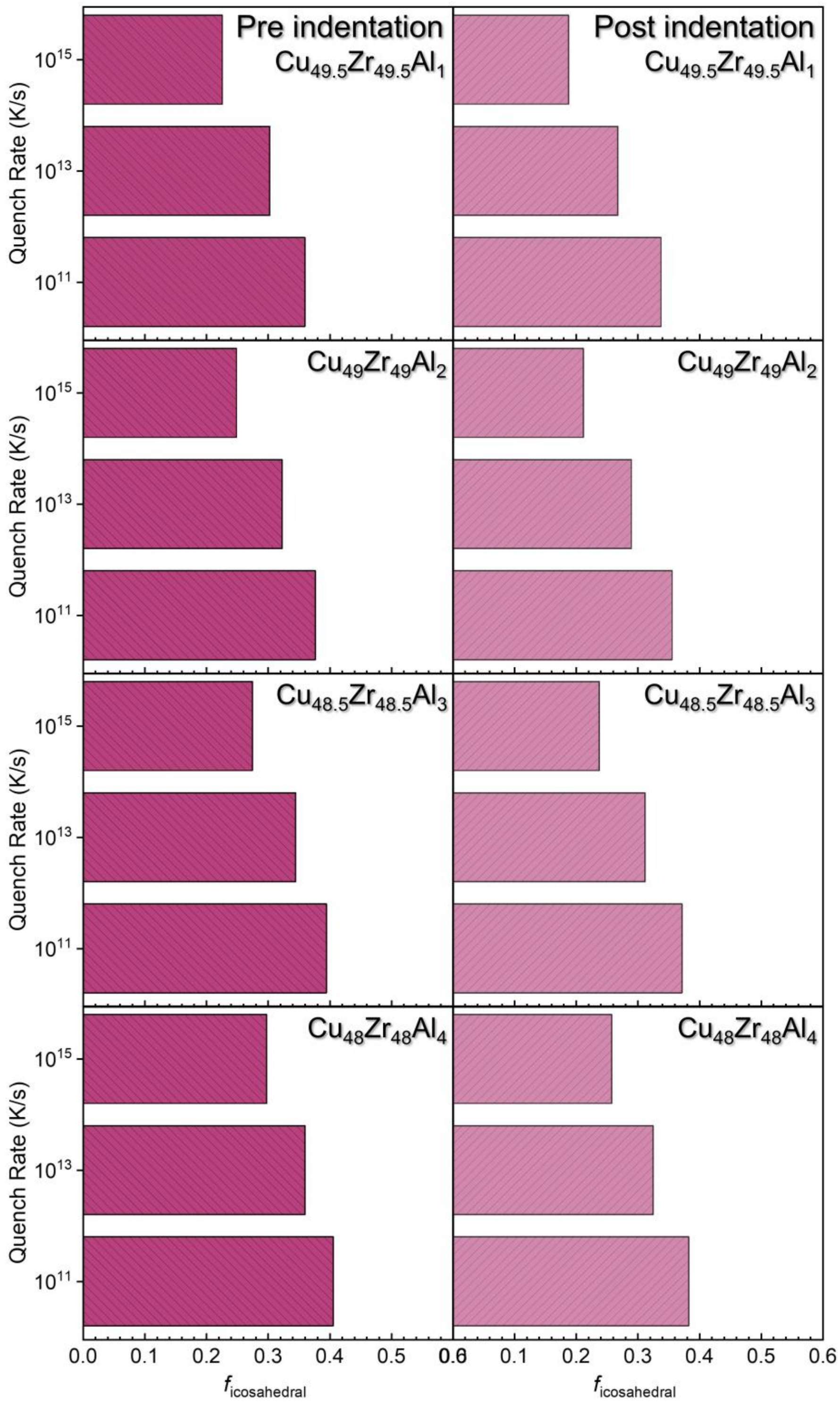

Figure S21 – Fraction of icosahedral motifs $f_{\text{icosahedral}}$ before and after indentation for $Cu_{50\text{-}z/2}Zr_{50\text{-}z/2}Al_z$ (z = 1-4 at.%) microalloying series at different quench rates (10^{11}, 10^{13}, 10^{15} K·s^{-1}).

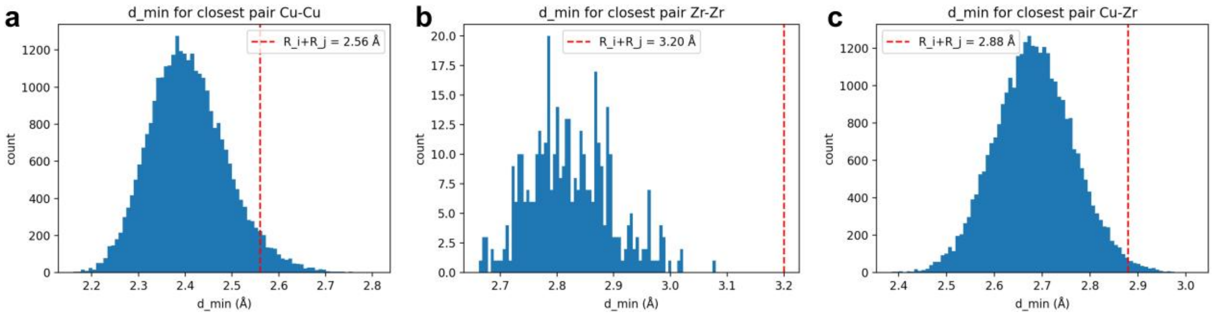

Figure S22 – Histograms of the minimum neighbour distance $d_{\min}$ for the closest pair in MD-quenched configurations: (a) Cu–Cu, (b) Zr–Zr, (c) Cu–Zr. The red dashed line marks the nominal hard-sphere contact distance $R_i + R_j$ from tabulated metallic radii (Cu = 1.28 Å, Zr = 1.60 Å), *i.e.*, 2.56 Å, 3.20 Å, and 2.88 Å for panels (a–c), respectively. In all cases the distributions substantially overlap and extend to $d_{\min} < R_i + R_j$, showing that interatomic separations in metallic glasses are not bounded by "metallic radii" (which are effective, environment-dependent sizes rather than impenetrable spheres).

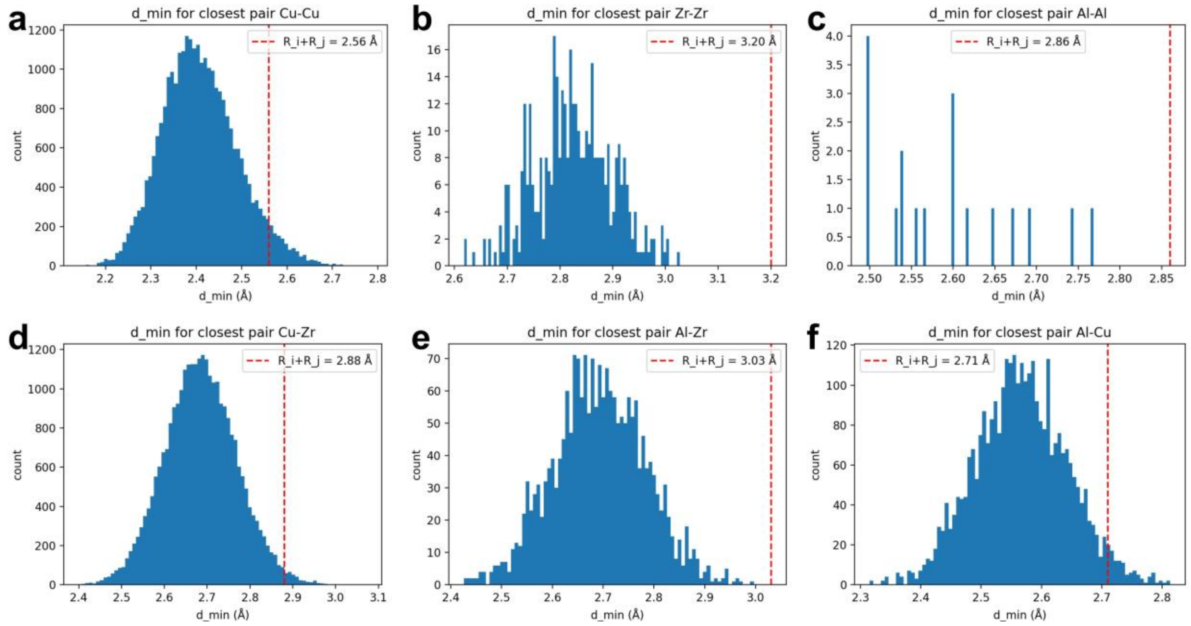

Figure S23 – Histograms of the minimum neighbour distance $d_{\min}$ for the closest pair in MD-quenched configurations: (a) Cu–Cu, (b) Zr–Zr, (c) Al–Al, (d) Cu–Zr, (e) Al–Zr, (f) Al–Cu. The red dashed line marks the nominal hard-sphere contact distance $R_i + R_j$ from tabulated metallic radii (Cu = 1.28 Å, Zr = 1.60 Å, Al = 1.43 Å), *i.e.*, 2.56 Å, 3.20 Å, 2.86 Å, 2.88 Å, 3.03 Å, and 2.71 Å for panels (a–f), respectively. In all cases the distributions substantially overlap and extend to $d_{\min} < R_i + R_j$, showing that interatomic separations in metallic glasses are not bounded by “metallic radii” (which are effective, environment-dependent sizes rather than impenetrable spheres).

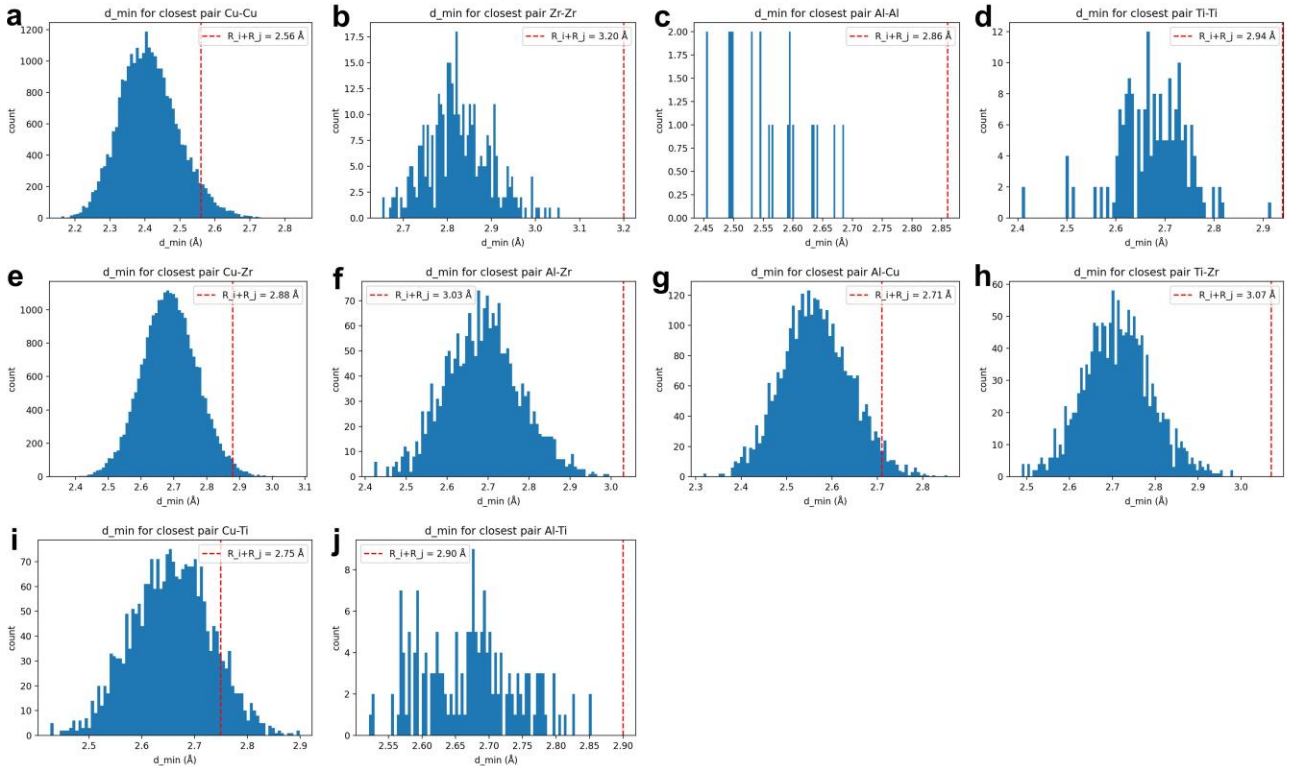

Figure S24 – Histograms of the minimum neighbour distance $d_{\min}$ for the closest pair in MD-quenched configurations: (a) Cu–Cu, (b) Zr–Zr, (c) Al–Al, (d) Ti–Ti, (e) Cu–Zr, (f) Al–Zr, (g) Al–Cu, (h) Ti–Zr, (i) Cu–Ti, (j) Al–Ti. The red dashed line marks the nominal hard-sphere contact distance $R_i + R_j$ from tabulated metallic radii (Cu = 1.28 Å, Zr = 1.60 Å, Al = 1.43 Å, Ti = 1.47 Å), *i.e.*, 2.56 Å, 3.20 Å, 2.86 Å, 2.94 Å, 2.88 Å, 3.03 Å, 2.71 Å, 3.07 Å, 2.75 Å and 2.90 Å for panels (a–f), respectively. In all cases the distributions substantially overlap and extend to $d_{\min} < R_i + R_j$, showing that interatomic separations in metallic glasses are not bounded by “metallic radii” (which are effective, environment-dependent sizes rather than impenetrable spheres).

Table S4 – Short-range order metrics from total RDFs for Cu-Zr-(Al/Ti) based metallic glasses for various quench rates at 300 K.

Alloy	Quench Rate (K/s)	g_1	g_{min}	S_2
$Cu_{40}Zr_{60}$	1×10^{11}	2.1559	0.262	0.0269
	1×10^{13}	1.9633	0.292	0.02157
	1×10^{15}	1.77459	0.337	0.0162
$Cu_{45}Zr_{55}$	1×10^{11}	2.20523	0.248	0.02913
	1×10^{13}	2.0533	0.279	0.02327
	1×10^{15}	1.89446	0.322	0.018
$Cu_{50}Zr_{50}$	1×10^{11}	2.25313	0.23916	0.0307
	1×10^{13}	2.13296	0.27025	0.026
	1×10^{15}	2.01001	0.30838	0.02132
$Cu_{60}Zr_{40}$	1×10^{11}	2.315	0.231	0.03159
	1×10^{13}	2.1754	0.262	0.02657
	1×10^{15}	2.04013	0.305	0.0216
$Cu_{40}Zr_{60}$	1×10^{11}	2.348	0.227	0.032
	1×10^{13}	2.19056	0.2595	0.02686
	1×10^{15}	2.05234	0.304	0.02179
$Cu_{49.5}Zr_{49.5}Al_1$	1×10^{11}	2.27831	0.23516	0.0317
	1×10^{13}	2.17403	0.26825	0.02707
	1×10^{15}	2.04995	0.30038	0.02242
$Cu_{49}Zr_{49}Al_2$	1×10^{11}	2.28931	0.23201	0.03261
	1×10^{13}	2.19233	0.264	0.02837
	1×10^{15}	2.08045	0.2972	0.024
$Cu_{48.5}Zr_{48.5}Al_3$	1×10^{11}	2.29897	0.2284	0.03328
	1×10^{13}	2.21048	0.2596	0.02983
	1×10^{15}	2.11481	0.29103	0.02554
$Cu_{48}Zr_{48}Al_4$	1×10^{11}	2.30278	0.22516	0.0347
	1×10^{13}	2.2374	0.25325	0.03193
	1×10^{15}	2.13995	0.28604	0.02824
$Cu_{47.5}Zr_{47.5}Al_5$	1×10^{11}	2.31897	0.22314	0.03528
	1×10^{13}	2.25048	0.25096	0.0327
	1×10^{15}	2.1641	0.28203	0.02984
$Cu_{45}Zr_{45}Al_5Ti_5$	1×10^{11}	2.35554	0.21117	0.03829
	1×10^{13}	2.32395	0.23314	0.03687
	1×10^{15}	2.28565	0.25321	0.03494

Table S5 – Summary of structure–mechanics metrics for Cu-Zr-based metallic glasses as a function of composition (rows) and quench rate (columns). Reported values are mean ± s.d. ($N = 5$ seeds). FVHI = $\sigma(V_{free})/\langle V_{free}\rangle$ from radical Voronoi volumes; H and E from Oliver–Pharr analysis of the unloading segment; ER = $(h_{max} - h_{res})/h_{max}$; $\rho_{pop\text{-}in}$ = pop-in count per nm depth after Savitzky–Golay smoothing; STZ count (nm^{-3}) and plastic-zone size from 3D voxel clustering (26-connectivity) of D^2min atoms thresholded by a far-field criterion ($\mu + 3\sigma$); $f_{icosahedral}$ is the fraction of icosahedral-type polyhedra pre/post indentation and $\Delta f_{icosahedral}$ is their difference. Overall, increasing compositional complexity (binary → ternary → quaternary) lowers FVHI, serration density, STZ density and plastic-zone volume while raising H, E and ER and suppressing quench-rate sensitivity.

Alloy	Quench Rate (K/s)	FVHI	Hardness (H, GPa)	Elastic Modulus (E, GPa)	Elastic recovery (ER)	$\rho_{pop\text{-}in}$ (nm^{-1})	STZ count (per nm^3)	Plastic zone size (nm^3)	$f_{icosahedral}$ (pre-indentation)	$f_{icosahedral}$ (post-indentation)	$\Delta f_{icosahedral}$
$Cu_{40}Zr_{60}$	1×10^{11}	0.260 ± 0.0032	7.62 ± 0.112	205.02 ± 2.87	0.405 ± 0.0036	7.22 ± 0.18	55 ± 6	24.15 ± 3.18	0.333	0.301	0.032
	1×10^{13}	0.286 ± 0.0017	6.81 ± 0.123	185.16 ± 2.96	0.384 ± 0.0037	8.24 ± 0.16	100 ± 5	38.34 ± 2.46	0.275	0.225	0.05
	1×10^{15}	0.309 ± 0.0023	6.05 ± 0.086	168.08 ± 1.72	0.360 ± 0.0034	9.41 ± 0.17	157 ± 6	58.04 ± 2.82	0.19	0.135	0.055
$Cu_{45}Zr_{55}$	1×10^{11}	0.252 ± 0.0041	7.92 ± 0.106	210.05 ± 2.13	0.415 ± 0.0037	6.90 ± 0.18	46 ± 8	21.28 ± 4.15	0.352	0.323	0.029
	1×10^{13}	0.274 ± 0.0022	7.21 ± 0.083	192.14 ± 2.00	0.397 ± 0.0025	7.81 ± 0.11	92 ± 6	33.64 ± 2.08	0.292	0.247	0.045
	1×10^{15}	0.295 ± 0.0031	6.54 ± 0.094	176.20 ± 1.89	0.372 ± 0.0037	8.79 ± 0.18	135 ± 4	50.84 ± 1.90	0.212	0.163	0.049
$Cu_{50}Zr_{50}$	1×10^{11}	0.244 ± 0.0037	8.16 ± 0.098	216.64 ± 1.97	0.424 ± 0.0039	6.61 ± 0.19	36 ± 6	18.21 ± 2.62	0.341	0.321	0.026
	1×10^{13}	0.262 ± 0.0036	7.50 ± 0.111	200.71 ± 2.67	0.412 ± 0.0033	7.42 ± 0.15	75 ± 6	28.65 ± 3.42	0.282	0.245	0.037
	1×10^{15}	0.285 ± 0.0028	6.91 ± 0.102	187.78 ± 2.04	0.394 ± 0.0041	8.39 ± 0.20	110 ± 6	42.24 ± 3.08	0.202	0.151	0.051
$Cu_{60}Zr_{40}$	1×10^{11}	0.237 ± 0.0025	8.45 ± 0.076	225.11 ± 1.52	0.435 ± 0.0040	6.10 ± 0.20	32 ± 4	16.82 ± 2.38	0.351	0.322	0.029
	1×10^{13}	0.255 ± 0.0022	7.65 ± 0.101	206.08 ± 2.41	0.421 ± 0.0030	6.91 ± 0.13	68 ± 5	25.54 ± 2.25	0.29	0.248	0.042
	1×10^{15}	0.274 ± 0.0030	7.01 ± 0.079	190.15 ± 1.58	0.402 ± 0.0031	7.94 ± 0.15	109 ± 3	41.54 ± 3.80	0.223	0.169	0.054
$Cu_{65}Zr_{35}$	1×10^{11}	0.235 ± 0.0034	8.48 ± 0.098	226.78 ± 1.97	0.437 ± 0.0041	6.03 ± 0.20	28 ± 6	14.51 ± 3.36	0.355	0.334	0.021
	1×10^{13}	0.253 ± 0.0037	7.69 ± 0.090	207.05 ± 2.16	0.422 ± 0.0027	6.81 ± 0.12	64 ± 5	23.82 ± 3.52	0.297	0.262	0.035
	1×10^{15}	0.274 ± 0.0024	7.06 ± 0.081	191.54 ± 1.62	0.402 ± 0.0032	7.89 ± 0.16	107 ± 5	40.11 ± 2.84	0.215	0.193	0.022
$Cu_{49.5}Zr_{49.5}Al_1$	1×10^{11}	0.243 ± 0.0021	8.33 ± 0.112	219.08 ± 2.46	0.428 ± 0.0026	6.44 ± 0.13	34 ± 3	17.22 ± 1.66	0.36	0.338	0.022
	1×10^{13}	0.259 ± 0.0033	7.68 ± 0.123	204.54 ± 2.59	0.418 ± 0.0027	7.15 ± 0.12	68 ± 4	26.31 ± 2.01	0.303	0.268	0.035
	1×10^{15}	0.280 ± 0.0038	7.13 ± 0.086	192.56 ± 1.72	0.403 ± 0.0020	8.11 ± 0.10	103 ± 4	39.26 ± 1.14	0.226	0.188	0.038
$Cu_{49}Zr_{49}Al_2$	1×10^{11}	0.242 ± 0.0024	8.51 ± 0.106	221.52 ± 2.13	0.433 ± 0.0028	6.26 ± 0.14	32 ± 3	16.43 ± 1.77	0.377	0.356	0.021
	1×10^{13}	0.257 ± 0.0018	7.90 ± 0.083	208.36 ± 1.76	0.423 ± 0.0018	6.96 ± 0.08	63 ± 2	25.15 ± 1.34	0.323	0.29	0.033
	1×10^{15}	0.276 ± 0.0026	7.43 ± 0.094	197.34 ± 1.89	0.410 ± 0.0022	7.80 ± 0.12	96 ± 4	37.43 ± 1.26	0.249	0.212	0.037
$Cu_{48.5}Zr_{48.5}Al_3$	1×10^{11}	0.241 ± 0.0037	8.69 ± 0.098	223.96 ± 1.97	0.437 ± 0.0026	6.12 ± 0.13	30 ± 3	15.60 ± 1.64	0.395	0.372	0.023
	1×10^{13}	0.256 ± 0.0020	8.18 ± 0.111	212.19 ± 2.33	0.429 ± 0.0024	6.74 ± 0.11	58 ± 3	23.56 ± 1.79	0.345	0.312	0.033
	1×10^{15}	0.271 ± 0.0023	7.79 ± 0.102	202.12 ± 2.04	0.417 ± 0.0024	7.56 ± 0.12	89 ± 4	34.62 ± 1.36	0.275	0.238	0.037
$Cu_{48}Zr_{48}Al_4$	1×10^{11}	0.240 ± 0.0026	8.86 ± 0.076	226.41 ± 1.52	0.442 ± 0.0020	5.92 ± 0.10	28 ± 3	14.81 ± 1.27	0.406	0.383	0.023
	1×10^{13}	0.252 ± 0.0035	8.45 ± 0.110	216.01 ± 2.11	0.434 ± 0.0022	6.52 ± 0.11	53 ± 3	22.38 ± 1.62	0.36	0.325	0.035
	1×10^{15}	0.264 ± 0.0034	8.06 ± 0.079	206.91 ± 1.58	0.424 ± 0.0018	7.32 ± 0.09	83 ± 3	32.82 ± 1.05	0.298	0.258	0.040
$Cu_{47.5}Zr_{47.5}Al_5$	1×10^{11}	0.239 ± 0.0024	8.99 ± 0.098	228.85 ± 1.97	0.446 ± 0.0026	5.80 ± 0.13	25 ± 3	14.68 ± 3.60	0.416	0.391	0.025
	1×10^{13}	0.250 ± 0.0031	8.61 ± 0.090	221.84 ± 1.89	0.437 ± 0.0019	6.31 ± 0.09	48 ± 2	21.43 ± 3.31	0.372	0.335	0.037
	1×10^{15}	0.260 ± 0.0030	8.25 ± 0.081	214.69 ± 1.62	0.431 ± 0.0019	7.02 ± 0.10	78 ± 3	31.19 ± 2.14	0.320	0.278	0.042
$Cu_{45}Zr_{45}Al_5Ti_5$	1×10^{11}	0.231 ± 0.0026	9.48 ± 0.129	236.47 ± 3.33	0.468 ± 0.0043	5.01 ± 0.15	21 ± 3	10.26 ± 2.16	0.560	0.543	0.017
	1×10^{13}	0.234 ± 0.0031	9.25 ± 0.153	234.87 ± 3.01	0.464 ± 0.0051	5.22 ± 0.13	35 ± 5	14.22 ± 2.24	0.541	0.520	0.021
	1×10^{15}	0.239 ± 0.0035	9.12 ± 0.176	229.41 ± 2.02	0.461 ± 0.0058	5.48 ± 0.18	49 ± 4	19.38 ± 4.11	0.527	0.500	0.027

*Residual indentation depth after unloading was extracted from post-indentation configurations. The elastic recovery ratio was computed using: $ER = \frac{h_{max}-h_{residual}}{h_{max}}$

References

[1] B.-J. Lee, M.I. Baskes, Second nearest-neighbor modified embedded-atom-method potential, Physical Review B, 62 (2000) 8564-8567.

[2] B.-J. Lee, J.-H. Shim, M.I. Baskes, Semiempirical atomic potentials for the fcc metals Cu, Ag, Au, Ni, Pd, Pt, Al, and Pb based on first and second nearest-neighbor modified embedded atom method, Physical Review B, 68 (2003) 144112.

[3] B.-J. Lee, M.I. Baskes, H. Kim, Y. Koo Cho, Second nearest-neighbor modified embedded atom method potentials for bcc transition metals, Physical Review B, 64 (2001) 184102.

[4] Y.-M. Kim, B.-J. Lee, M.I. Baskes, Modified embedded-atom method interatomic potentials for Ti and Zr, Physical Review B, 74 (2006) 014101.

[5] B.A. Hatt, J.A. Roberts, G.I. Williams, Occurrence of the Meta-stable Omega Phase in Zirconium Alloys, Nature, 180 (1957) 1406-1406.

[6] P. Duwez, Allotropic transformation in titanium-zirconium alloys, in, California Inst. of Tech., Pasadena, CA (United States). Mechanical …, 1952.

[7] J.D. Fast, The transition point diagram of the zirconium-titanium system, Recueil des Travaux Chimiques des Pays-Bas, 58 (1939) 973-983.

[8] R. Sandler, Structure of Spongy Ti-Zr Alloys Obtained by Thermal Reduction With Mg, Izv. Akad. Nauk SSSR, Met., (1975) 30-33.

[9] H.W. Sheng, W.K. Luo, F.M. Alamgir, J.M. Bai, E. Ma, Atomic packing and short-to-medium-range order in metallic glasses, Nature, 439 (2006) 419-425.

[10] G. Kumar, P. Neibecker, Y.H. Liu, J. Schroers, Critical fictive temperature for plasticity in metallic glasses, Nature Communications, 4 (2013) 1536.
